# Correction: Anwer et al. Molecular and Morphological Characterization of *Exserohilum turcicum* (Passerini) Leonard and Suggs Causing Northern Corn Leaf Blight of Maize in Bihar. *Bioengineering* 2022, *9*, 403

**DOI:** 10.3390/bioengineering11040361

**Published:** 2024-04-10

**Authors:** Md Arshad Anwer, Ram Niwas, Tushar Ranjan, Shyam Sundar Mandal, Mohammad Ansar, Jitendra Nath Srivastava, Jitesh Kumar, Khushbu Jain, Neha Kumari, Aditya Bharti

**Affiliations:** 1 Department of Plant Pathology, Bihar Agricultural University, Sabour 813210, Bhagalpur, India; ansar.pantversity@gmail.com (M.A.); j.n.srivastava1971@gmail.com (J.N.S.); neha.k1392@gmail.com (N.K.); adityabharti0806@gmail.com (A.B.); 2 Department of Molecular Biology and Genetic Engineering, Bihar Agricultural University, Sabour 813210, Bhagalpur, India; mail2tusharranjan@gmail.com (T.R.); jitesh1jan@gmail.com (J.K.); khushbu3aug@gmail.com (K.J.); 3 Department of Plant Breeding and Genetics, Bihar Agricultural University, Sabour 813210, Bhagalpur, India; maizebreederbau@gmail.com

## Error in Figure

In the original publication [1], there were mistakes in Figures 1 and 3 as published. The duplication of microscopy data was a mistake made by authors during the preparation of the manuscript. The corrected Figure 1 and Figure 3 appear below.

**Figure 1:**
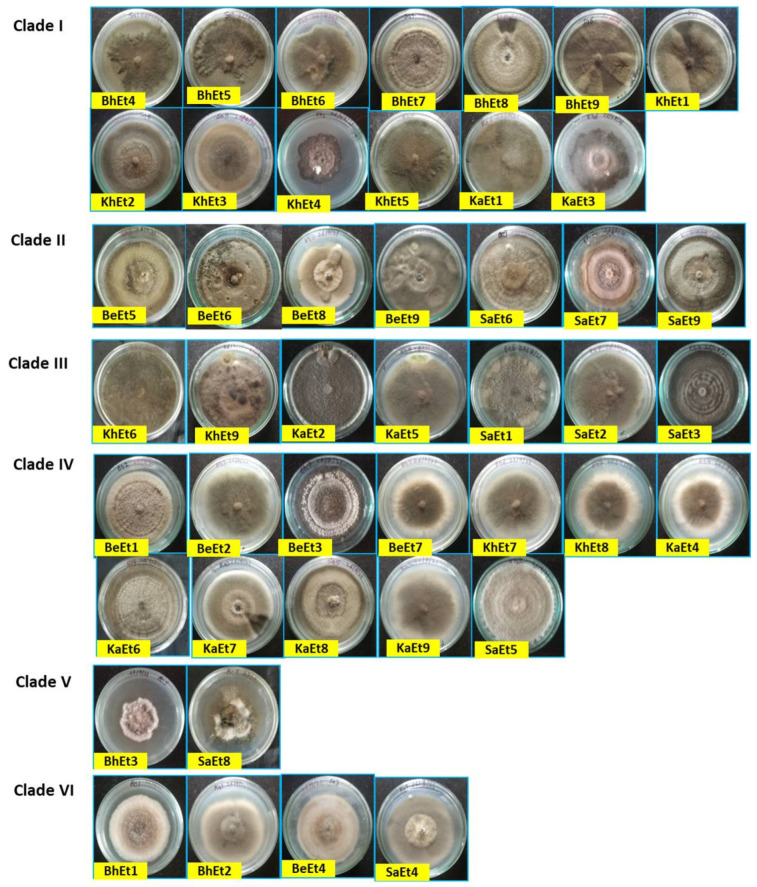
Cultural and morphological variability of different isolates of *E. turcicum* on PDA (front view) 10 days after inoculation.

**Figure 3:**
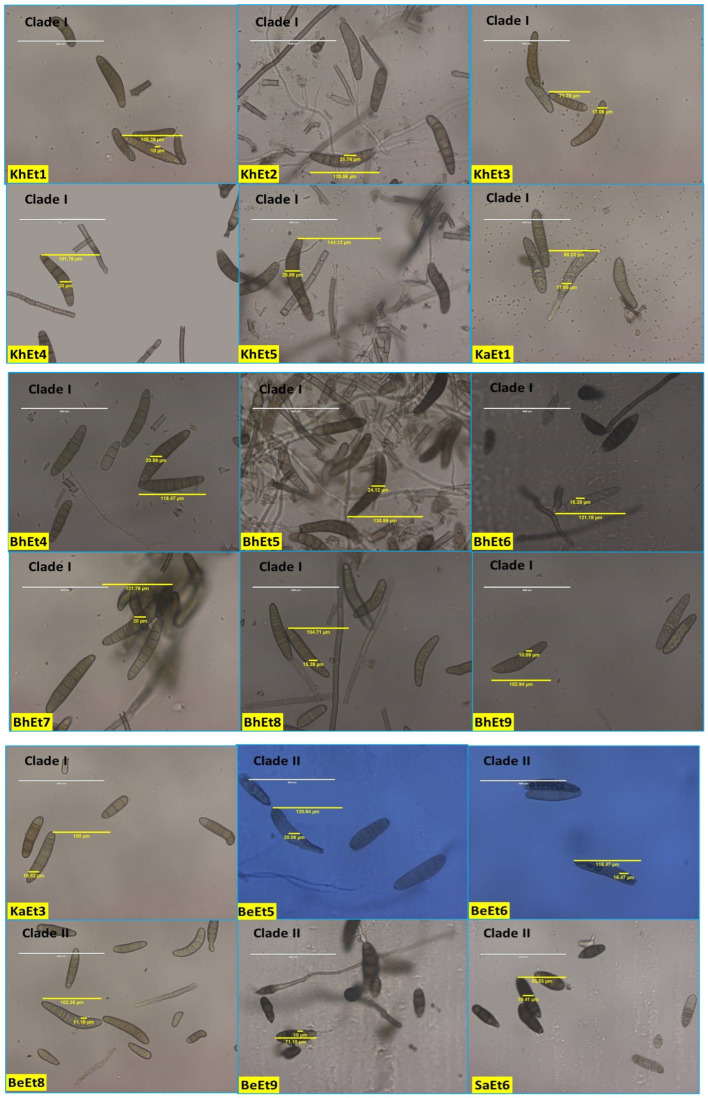

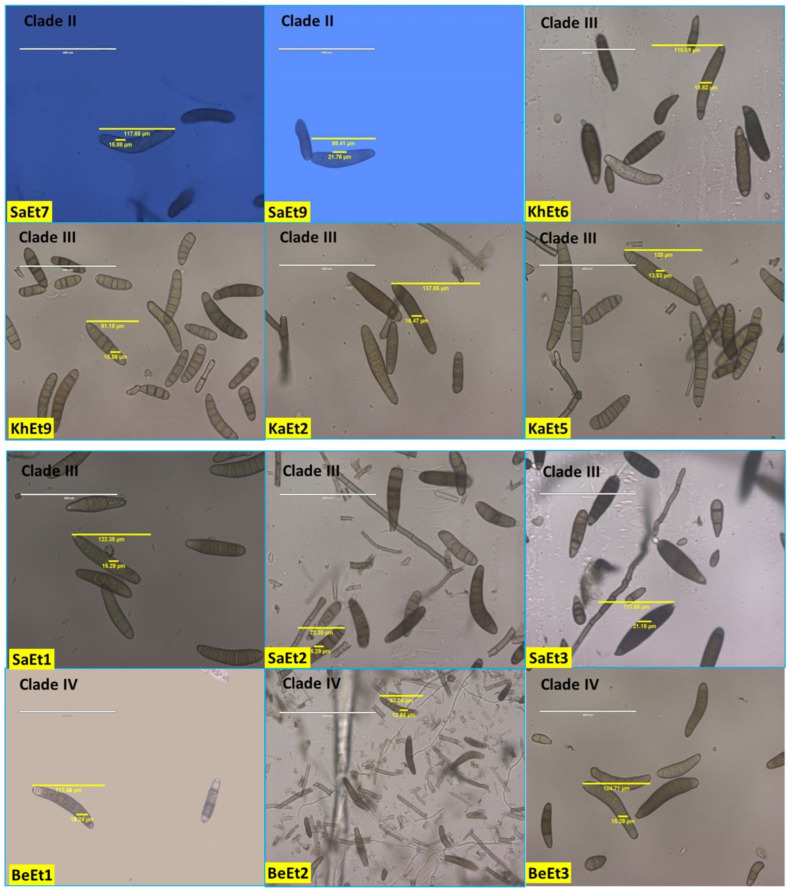

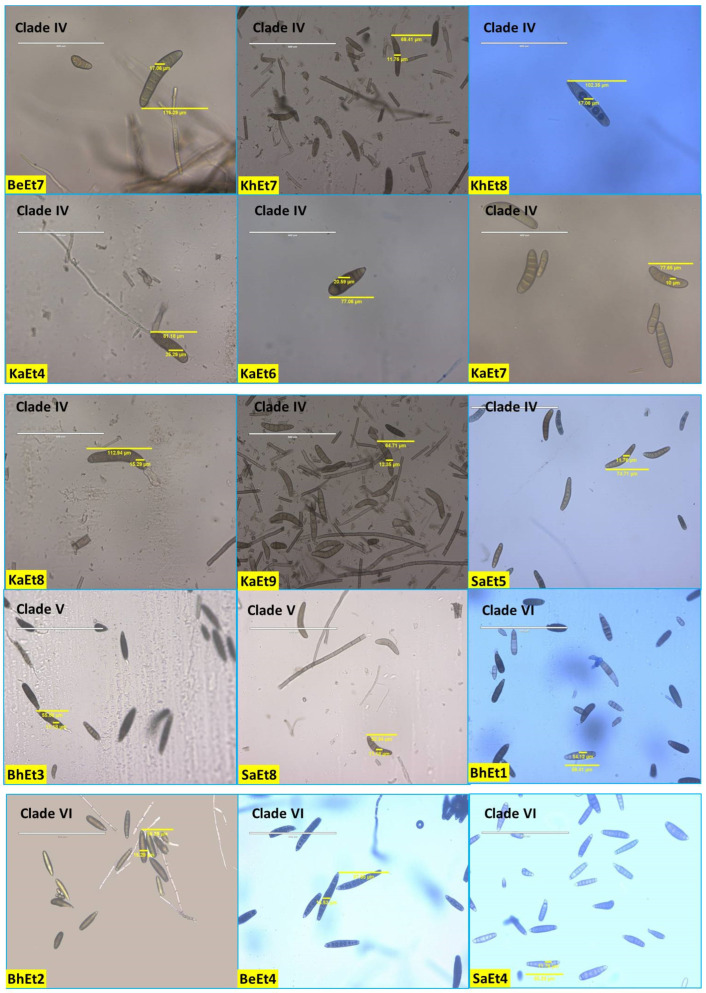
Conidial variability of different isolates of *E. turcicum* causing NCLB.

The authors state that the scientific conclusions are unaffected. This correction was approved by the Academic Editor. The original publication has also been updated.
