# Supplementary material for: Molecular and Morphological Characterization of Exserohilum turcicum (Passerini) Leonard and Suggs Causing Northern Corn Leaf Blight of Maize in Bihar
Source: Bioengineering (Basel). 2022 Aug 19;9(8):403. doi: 10.3390/bioengineering9080403 (PMC9405478; doi:10.3390/bioengineering9080403)
Supplement: Supplementary file 1 [file bioengineering-09-00403-s001.zip › bioengineering-1870517-supplementary.pdf]

Figure S1. Multiple sequence alignment of beta-tubulin gene of all 45 E. turcicum isolates using Clustal Omega

```
Reference sequence (1): BhEt_3
Identities normalised by aligned length.
Colored by: identity


                     cov    pid     1 [        .         .         .         :         .         .         . 80
  1 BhEt_3         100.0% 100.0%     --------------------------------------------------------------------------------
  2 BeEt_2          60.7%  19.0%     ------------------------CTCAGTGAACTCCATCTCGTCCATACCCTCACCATGTATACCAATGCA-AGAAAGC
  3 BeEt_1          60.7%  19.7%     -------------------------------------------------CTCACCATGTATACCAATGCA-AGAAAGC
  4 KhEt_1          82.5%  23.5%     -----------------------CCTCAGTGACTCCATCTCGGTCCATACCCTCACCAGTGTACCAATGCAA--GAAAGC
  5 BeEt_9          94.5%  24.6%     -GCGAAAACTGGTGTGGAAATGGCCTCAGTGTACTCCATCTCGTCCATACCCTCACCAGTGTACCAATGCA--AGAAAGC
  6 SaEt_7          95.0%  23.9%     ---GGGGTTATGCTTGGGATCAGCCTCAGTGCACTCCATCTCGTCCATACCCTCACCAGTGTACCAATGCAAGCATAAGC
  7 SaEt_6          92.6%  24.3%     ---GGGGTTATGCTTGGGATCAGCCTCAGTGCACTCCATCTCGTCCATACCCTCACCAGTGTACCAATGCAAGCATAAGC
  8 SaEt_9          93.9%  23.7%     --CCCCCCCATGCCCG-CGATCAGCCTCAGTGACTCCATCTCGTCCATACCCTCACCAGTGTACCAATGCA--AGAAAGC
  9 BeEt_8          93.2%  22.9%     ----CAATTAGCTTGGGAATCAGCCTCAGTGAACTCCATCTCGTCCATACCCTCACCAGTGTACCAATGCAA--GAAAGC
 10 BeEt_6          82.5%  22.4%     ------CGTATAGCTTGGATCAG-CCTCAGTGACTCCATCTCGTCCATACCCTCACCAGTGTACCAATGCAA-GCAAAGC
 11 BeEt_5          92.9%  24.2%     ------CGTATAGCTTGGATCAG-CCTCAGTGACTCCATCTCGTCCATACCCTCACCAGTGTACCAATGCAA-GCAAAGC
 12 BhEt_7          82.5%  24.1%     GTTGGTGGCCACGACTATCGATCGGCTCAGTGACTCCATCTCGTCCATGCCCTCACCAGTGTACCAATGCAA-GAAAG-C
 13 BhEt_6          84.6%  23.2%     GTTGGTGGCCACGACTATCGATCGGCTCAGTGACTCCATCTCGTCCATGCCCTCACCAGTGTACCAATGCAA-GAAAG-C
 14 BhEt_5          88.4%  24.2%     GTTGGTGGCCACGACTATCGATCGGCTCAGTGACTCCATCTCGTCCATGCCCTCACCAGTGTACCAATGCAA-GAAAG-C
 15 BhEt_4          90.9%  24.9%     GTTGGTGGCCACGACTATCGATCGGCTCAGTGACTCCATCTCGTCCATGCCCTCACCAGTGTACCAATGCAA-GAAAG-C
 16 KhEt_2          80.3%  24.2%     -----------------------GCCTCGGTGACTCCATCTCGTCCATACCCTCACCAGTGTACCAATGCAA-GAAAG-C
 17 KaEt_1          61.0%  24.9%     ------------------TTCCTCAGTGAACTCCATTCTCGGTCCATACCCTCACCAGTGTACCAATGCAA-GAAAG-C
 18 KhEt_5          81.4%  25.4%     ------------------TCCTCAGTGAACTCCATCTTCGGTCCATACCCTTCACCAGTGTACCAATGCAA-GAAAG-C
 19 KhEt_3          81.1%  24.6%     ----------------GCCTTCAGTGGAACTCCGTCTTCGGTCCATACCCTTCACCAGTGTACCAATGCAA-GAAAG-C
 20 KhEt_4          82.5%  25.1%     -------------------------TCAGTGAACTCCATCTCGTCCATACCCTCACCAGTGTACCAATGCAG-GAAAG-C
 21 KaEt_3          68.2%  24.9%     ----------------------CCTCAGTGGAACTCCATCTCGTCCATACCCTCACCAGTGTACCAATGCAG-GAAAG-C
 22 BhEt_9          57.5%  24.6%     -----------------------CTCAGTTGAACTCCATCTCGTCCATACCCTCACCAGTGTACCAATGCAA-GAAAG-C
 23 BhEt_8          59.9%  25.3%     -----------------------CTCAGTTGAACTCCATCTCGTCCATACCCTCACCAGTGTACCAATGCAA-GAAAG-C
 24 KaEt_6          82.7%  23.9%     --------------------------CGGTGCACTCCGTCTCGTCCATACCCTCACCTTTGTACCAATGCAA-GATAGCC
 25 SaEt_5          91.0%  23.4%     --GTGGTTTGCACGCTTCGGCTCGACTCAGTGACTCCATCTCGTCCATACCCTCACCAGTGTACCAATGCAA-GAAAGCC
 26 KaEt_7          82.2%  24.1%     -----------------------GCCTCAGTGACTCCATCTCGTCCATACCCTCACCAGTGTACCAATGCAA-GAAAGCC
 27 KaEt_4          82.4%  24.1%     ------------------------CCTCAGTGACTCCATCTCGTCCATACCCTCACCAGTGTACCAATGCAA-GAAAGCC
 28 KaEt_9          77.2%  24.2%     ----------------------GCCTCAGTGCACTCCATCTCGTCCATACCCTCACCAGTGTACCAATGCAA-GAAAGCC
 29 KaEt_8          81.9%  24.1%     -----------------------GCCTCAGTGCACTCCATCTCGTCCATACCCTCACCAGTGTACCAATGCAA-GAAAGCC
 30 BeEt_7          81.8%  23.9%     ---------------------GGCCTCAGTTGACTCCATCTCGGTCCATACCCTCACCAGTGTACCAATGCAA-GAAAGCC
 31 BeEt_3          82.1%  23.7%     ----------------------CCTCAGGTGAACTCCATCTCGTCCATACCCTCACCAGTGTACCAATGCAA-GAAAGCC
 32 KhEt_8          82.2%  23.8%     -----------------------CCTCAGGTGACTCCATCTCGTCCATACCCTCACCAGTGTACCAATGCAA-GAAAGCC
 33 KhEt_7          78.5%  23.9%     -----------------------CCTCAGGTGACTCCATCTCGTCCATACCCTCACCAGTGTACCAATGCAA-GAAAGCC
 34 BhEt_2          61.8%  22.7%     ---------------------------TCTGAACTCCATCTCGTCCATACCCTCACCAGTGTACCAATGCAT-GAA-AGC
 35 BhEt_1          52.4%  18.6%     ---------------------------TCTGAACTCCATCTCGTCCATACCCTCACCAGTGTACCAATGCAT-GAA-AGC
 36 BeEt_4          83.5%  26.0%     -----------------------------TGAACTCCGGCTCGTCCATACCCTCACCAGTGG--ACCGTGCA-TGAAGGC
 37 SaEt_3          81.9%  25.0%     ----------------------GCCTTTTGAATCCATCTTCGTCCATACCCTTCACCAGTGTACCAATGCAA-GAAAGCC
 38 KhEt_9          82.7%  25.2%     --------------------------TCAGTGACTCCATCTCAGCCATACCCTCACCAGTGTACCAGTGCAA-GAAAG-C
 39 SaEt_4          82.7%  25.9%     --------------------------TCAGTGACTCCATCTCGTCCATACCCTCACCAGTGTACCAATGCAA-GAAAG-C
 40 SaEt_8          91.0%  25.3%     --------CTGCAGGCTGGATCGGCCTCAGTGACTCCATCTCGTCCATACCCTCACCAGTGTACCAATGCAA-GAAAG-C
 41 KaEt_5          91.7%  25.0%     ---------CATCAGCTGGATCGGCCTCAGTGACTCCATCTCGTCCATACCCTCACCAGTGTACCAATGCAA-GAAAG-C
 42 KaEt_2          82.2%  25.9%     -----------------------GCCTCAGTGACTCCATCTCGTCCATACCCTCACCAGTGTACCAATGCAA-GAAAG-C
 43 KhEt_6          82.5%  25.9%     -------------------------CTCAGTGACTCCATCTCGTCCATACCCTCACCAGTGTACCAATGCAA-GAAAG-C
 44 SaEt_2          70.8%  26.0%     ------------------------CTCAGGTGACTCCATCTCGTCCATACCCTCACCAGTGTACCAATGCAA-GAAAG-C
 45 SaEt_1          82.4%  25.9%     ------------------------CTCAGGTGACTCCATCTCGTCCATACCCTCACCAGTGTACCAATGCAA-GAAAG-C
    consensus/100%                   ................................................................................
    consensus/90%                    ...........................ssssssCssssssssssCssssCCCTCACCAGTGTACCAATGCAs.suAAu.C
    consensus/80%                    .........................sssssssACTCCuTCTCGTCCATACCCTCACCAGTGTACCAATGCAA.uuAAu.C
    consensus/70%                    ........................ssssussuACTCCATCTCGTCCATACCCTCACCAGTGTACCAATGCAA.GAAAG.C

                     cov    pid    81         .         1         .         .         .         .         :         . 160
  1 BhEt_3         100.0% 100.0%     ------------CATTTCCGGGAGCGATGGTCAGGCACTGGTCGACAGGTCCTCGACGTCGTCCGCCGTGAGGCCGAGGG
  2 BeEt_2          60.7%  19.0%     CTTGCGTCGGAACATGGCAGTGAACTGCTCACCAACACGCTTGAAGAGCTCCTGAATAGAAGTGGAGTTTCCGATGAAGG
  3 BeEt_1          60.7%  19.7%     CTTGCGTCGGAACATGGCAGTGAACTGCTCACCAACACGCTTGAAGAGCTCCTGAATAGAAGTGGAGTTTCCGATGAAGG
  4 KhEt_1          82.5%  23.5%     CTTGCGTCGGAACATGGCAGTGAACTGGTCACCGACACGCTTGAAAAGCTCCTGGATGGAGGTGGAGTTTCCGACGA-GG
  5 BeEt_9          94.5%  24.6%     CTTGCGACGGAACATGACGGTGAACTGCTCACCGACACGCTTGAAGAGCTCCTGGATGGAGGTGGAGTTACCGATGAAGG
  6 SaEt_7          95.0%  23.9%     CTTGCGACGGAACATGACGGTGAACTGCTCACCGACACGCTTGAAGAGCTCCTGGATGGAGGTGGAGTTACCGATGAAGG
  7 SaEt_6          92.6%  24.3%     CTTGCGACGGAACATGACGGTGAACTGCTCACCGACACGCTTGAAGAGCTCCTGGATGGAGGTGGAGTTACCGATGAAGG
  8 SaEt_9          93.9%  23.7%     CTTGCGACGGAACATGACGGTGAACTGCTCACCGACACGCTTGAAGAGCTCCTGGATGGAGGTGGAGTTACCGATGAAGG
  9 BeEt_8          93.2%  22.9%     CTTGCGACGGAACATGACGGTGAACTGCTCACCGACACGCTTGAAGAGCTCCTGGATGGAGGTGGAGTTACCGATGAAGG
 10 BeEt_6          82.5%  22.4%     CTTGCGACGGAACATGACGGTGAACTGCTCACCGACACGCTTGAAGAGCTCCTGGATGGAGGTGGAGTTACCGATGAAGG
 11 BeEt_5          92.9%  24.2%     CTTGCGACGGAACATGACGGTGAACTGCTCACCGACACGCTTGAAGAGCTCCTGGATGGAGGTGGAGTTACCGATGAAGG
 12 BhEt_7          82.5%  24.1%     CTTGCGCCTGAACATGGCAGTGAACTGGTCGCCGACACGCTTGAAGAGCTCCTGGATGGAGGTCGAGTTACCGACGAAGG
 13 BhEt_6          84.6%  23.2%     CTTGCGCCTGAACATGGCAGTGAACTGGTCGCCGACACGCTTGAAGAGCTCCTGGATGGAGGTCGAGTTACCGACGAAGG
 14 BhEt_5          88.4%  24.2%     CTTGCGCCTGAACATGGCAGTGAACTGGTCGCCGACACGCTTGAAGAGCTCCTGGATGGAGGTCGAGTTACCGACGAAGG
 15 BhEt_4          90.9%  24.9%     CTTGCGCCTGAACATGGCAGTGAACTGGTCGCCGACACGCTTGAAGAGCTCCTGGATGGAGGTCGAGTTACCGACGAAGG
 16 KhEt_2          80.3%  24.2%     CTTGCGCCGGAACATGGCAGTGAACTGGTCACCGACACGCTCGGAGAGCTCCTGGATGGAGGTCGAGTTACCGACAAAGG
 17 KaEt_1          61.0%  24.9%     CTTGCGCCTGAACATGGCAGTGAACTGGTCACCGACACGCTTGAAGAGCTCCTGGATGGAGGTCGAGTTACCGACGAAGG
 18 KhEt_5          81.4%  25.4%     CTTGTGCCTGAACATGGCAGTGAACTGGTCACCGACACGCTTGAAGAGCTCCTGGATGGAGGTCGAGTTACCGACGAAGG
 19 KhEt_3          81.1%  24.6%     CTTGCGCCTGAACATGGCAGTGAACTGGTCACCGACACGCTTGAAGAGCTCCTGGATGGAGGTCGAGTTACCGACGAAGG
```

```
 20 KhEt_4          82.5%  25.1%      CTTGCGCCTGAACATGGCCGTGAACTGGTCACCGACACGCTAGAAGAGCTCCTGGATGGAGGTCGAGTTACCGACCAAGG
 21 KaEt_3          68.2%  24.9%      CTTGCGCCTGAACATGGCAGTGAACTGGTCACCGACACGCTCGAAGAGCTCCTGGATGGAGGTCGAGTTACCGACGAAGG
 22 BhEt_9          57.5%  24.6%      CTTGCGCCTGAACATGGCAGTGAACTGGTCACCGACACGCTTGAAGAGCTCCTGGATGGAGGTCGAGTTACCGACGAAGG
 23 BhEt_8          59.9%  25.3%      CTTGCGCCTGAACATGGCAGTGAACTGGTCACCGACACGCTTGAAGAGCTCCTGGATGGAGGTCGAGTTACCGACGAAGG
 24 KaEt_6          82.7%  23.9%      -TTGCGTCGGAACATAGCAGTAAACTGCTCGCCAACACGCTTGAAAAGCTCCTGGATAGAGGTGGAGTTTCCAATAAAGG
 25 SaEt_5          91.0%  23.4%      T-TGGAACGGAACATAGCAGTAAACTGCTCGCCAACACGCTTGAAAAGCTCCTGGATAGAGGTGGAGTTTCCAATAAAGG
 26 KaEt_7          82.2%  24.1%      T-TGCGTCGGAACATAGCAGTAAACTGCTCGCCAACACGCTTGAAAAGCTCCTGGATAGAGGTGGAGTTTCCAATAAAGG
 27 KaEt_4          82.4%  24.1%      T-TGCGTCGGAACATAGCAGTAAACTGCTCGCCAACACGCTTGAAAAGCTCCTGGATAGAGGTGGAGTTTCCAATAAAGG
 28 KaEt_9          77.2%  24.2%      TTGTTGTCGGAACATAGCAGTAAACTGCTCGCCAACACGCTTGAAAAGCTCCTGGATAGAGGTGGAGTTTCCAATAAAGG
 29 KaEt_8          81.9%  24.1%      TTGTTGTCGGAACATAGCAGTAAACTGCTCGCCAACACGCTTGAAAAGCTCCTGGATAGAGGTGGAGTTTCCAATAAAGG
 30 BeEt_7          81.8%  23.9%      T-TGCGTCGGAACATAGCAGTAAACTGCTCGCCAACACGCTTGAAAAGCTCCTGGATAGAGGTGGAGTTTCCAATAAAGG
 31 BeEt_3          82.1%  23.7%      T-TGCGTCGGAACATAGCAGTAAACTGCTCGCCAACACGCTTGAAAAGCTCCTGGATAGAGGTGGAGTTTCCAATAAAGG
 32 KhEt_8          82.2%  23.8%      T-TGCGTCGGAACATAGCAGTAAACTGCTCGCCAACACGCTTGAAAAGCTCCTGGATAGAGGTGGAGTTTCCAATAAAGG
 33 KhEt_7          78.5%  23.9%      T-TGCGTCGGAACATAGCAGTAAACTGCTCGCCAACACGCTTGAAAAGCTCCTGGATAGAGGTGGAGTTTCCAATAAAGG
 34 BhEt_2          61.8%  22.7%      CTTGCTCCTGAACATGGCAGTGAACTGGTCAGCGACACGCTTGAACAGCTCCTGGATGGAGGTCGAGTTACCAACGAAGG
 35 BhEt_1          52.4%  18.6%      CTTGCTCCTGAACATGGCAGTGAACTGGTCAGCGACACGCTTGAACAGCTCCTGGATGGAGGTCGAGT------------
 36 BeEt_4          83.5%  26.0%      CTTGCGGCTGACCATGGGCCTGAACTGGTCAGCGACACGCTTGAACAGCTCCTGGATGGAGGTCGAGTTACCAACGAAGG
 37 SaEt_3          81.9%  25.0%      TTGAATACTGAACATGGCAGTGAACTGGTCACCGACACGCTTGAATATCTCCTGAATGGAGGTCGAGTTACCGACGAAGG
 38 KhEt_9          82.7%  25.2%      CTTGCGCCTGAACATGGCAGTGAACTGGTCACCAACACGCTTGAAGAGCTCCTGGATGGAGGTCGAGTTACCGACGAAGG
 39 SaEt_4          82.7%  25.9%      CTTGCGCCTGAACATGGCAGTGAACTGGTCACCGACACGCTTGAACAGCTCCTGGATGGAGGTCGAGTTACCAACGAAGG
 40 SaEt_8          91.0%  25.3%      CTTGCGCCTGAACATGGCAGTGAACTGGTCACCGACACGCTTGAAGAGCTCCTGAATGGAGGTCGAGTTACCGACGAAGG
 41 KaEt_5          91.7%  25.0%      CTTGCGCCTGAACATGGCAGTGAACTGGTCACCGACACGCTTGAAGAGCTCCTGAATGGAGGTCGAGTTACCGACGAAGG
 42 KaEt_2          82.2%  25.9%      CTTGCGCCTGAACATGGCAGTGAACTGGTCACCGACACGCTTGAACAGCTCCTGGATCGAGGTCGAGTTACCGACGAAGG
 43 KhEt_6          82.5%  25.9%      CTTGCGCCTGAACATGGCAGTGAACTGGTCACCGACACGCTTGAACAGCTCCTGGATCGAGGTCGAGTTACCGACGAAGG
 44 SaEt_2          70.8%  26.0%      CTTGCGCCTGAACATGGCAGTGAACTGGTCACCGACACGCTTGAACAGCTCCTGGATCGAGGTCGAGTTACCGACGAAGG
 45 SaEt_1          82.4%  25.9%      CTTGCGCCTGAACATGGCAGTGAACTGGTCACCGACACGCTTGAACAGCTCCTGGATCGAGGTCGAGTTACCGACGAAGG
    consensus/100%                    ............CATssssssuAuCsussssssuuCACsssssuAsAssTCCTsuAssssusssGsss............
    consensus/90%                     s.TGsusCsGAACATuuCuGTuAACTGsTCuCCuACACGCTTGAAsAGCTCCTGuATuGAGGTsGAGTTsCCuAsuAAGG
    consensus/80%                     sTTGCGsCsGAACATuGCuGTuAACTGsTCuCCuACACGCTTGAAsAGCTCCTGGATuGAGGTsGAGTTsCCuAsuAAGG
    consensus/70%                     CTTGCGsCsGAACATGGCAGTGAACTGsTCuCCGACACGCTTGAAuAGCTCCTGGATuGAGGTsGAGTTsCCuAsGAAGG

                      cov    pid  161          .         .         .         2         .         .         .         . 240
  1 BhEt_3         100.0% 100.0%      CTGCGACTGCCTCCAGGGCTTCCAGATCACCCACTCCCTCGGTGGTGGAACCGGTGCCGGTATGGGTAC----TCTCCTC
  2 BeEt_2          60.7%  19.0%      TCGAAGACATCGTAAGTCCTCGGGG---------------AGGGATGGCACAAAGGGCTGTCTGGATGTTGTTGGGAATC
  3 BeEt_1          60.7%  19.7%      TCGAAGACATCGTAAGTCCTCGGGG---------------AGGGATGGCACAAAGGGCTGTCTGGATGTTGTTGGGAATC
  4 KhEt_1          82.5%  23.5%      TGGAGGACATCTTGAGGCCCCGGGG---------------AGAAACGGACCAAAGGGGGGTCGGGAGTTTTTTGGGGATC
  5 BeEt_9          94.5%  24.6%      TAGAAGACATCTTGAGGCCACGGGG---------------AGGAATGGCGCAGAGAGCGGTCTGGATGTTGTTGGGAATC
  6 SaEt_7          95.0%  23.9%      TAGAAGACATCTTGAGGCCACGGGG---------------AGGAATGGCGCAGAGAGCGGTCTGGATGTTGTTGGGAATC
  7 SaEt_6          92.6%  24.3%      TAGAAGACATCTTGAGGCCACGGGG---------------AGGAATGGCGCAGAGAGCGGTCTGGATGTTGTTGGGAATC
  8 SaEt_9          93.9%  23.7%      TAGAAGACATCTTGAGGCCACGGGG---------------AGGAATGGCGCAGAGAGCGGTCTGGATGTTGTTGGGAATC
  9 BeEt_8          93.2%  22.9%      TAGAAGACATCTTGAGGCCACGGGG---------------AGGAATGGCGCAGAGAGCGGTCTGGATGTTGTTGGGAATC
 10 BeEt_6          82.5%  22.4%      TAGAAGACATCTTGAGGCCACGGGG---------------AGGAATGGCGCAGAGAGCGGTCTGGATGTTGTTGGGAATC
 11 BeEt_5          92.9%  24.2%      TAGAAGACATCTTGAGGCCACGGGG---------------AGGAATGGCGCAGAGAGCGGTCTGGATGTTGTTGGGAATC
 12 BhEt_7          82.5%  24.1%      TGGAGGACATCTTGAGGCCGCGGGG---------------AGGAACGGAGCAGAGGGCGGTCTGGACGTTGTTGGGGATC
 13 BhEt_6          84.6%  23.2%      TGGAGGACATCTTGAGGCCGCGGGG---------------AGGAACGGAGCAGAGGGCGGTCTGGACGTTGTTGGGGATC
 14 BhEt_5          88.4%  24.2%      TGGAGGACATCTTGAGGCCGCGGGG---------------AGGAACGGAGCAGA--------------------GGGATC
 15 BhEt_4          90.9%  24.9%      TGGAGGACATCTTGAGGCCGCGGGG---------------AGGAACGGAGCAGAGGGCGGTCTGGACGTTGTTGGGGATC
 16 KhEt_2          80.3%  24.2%      TCGAGGACATCTTGAGGCCGCGAGG---------------AGGAACGGAGCAGAGAGCGGTCTGGACGTTGTTGGGGATC
 17 KaEt_1          61.0%  24.9%      TGGAGGACATCTTGAGGCCGCGGGG---------------AGGAACGGAGCAGAGGGCGGTCTGGACGTTGTTGGGGATC
 18 KhEt_5          81.4%  25.4%      TGGAGGACATCTTGAGGCCGCGGGG---------------AGGAACGGAGCAGAGGGCGGTCTGGACGTTGTTGGGGATC
 19 KhEt_3          81.1%  24.6%      TGGAGGACATCTTGAGGCCGCGGGG---------------AGGAACGGAGCAGAGGGCGGTCTGGACGTTGTTGGGGATC
 20 KhEt_4          82.5%  25.1%      TGGAGGACATCTTGAGGCCGCGGGG---------------AGGAACGGAGCATAGGGCGGTCTGGACGTTGTTGGGGATC
 21 KaEt_3          68.2%  24.9%      TGGAGGACATCTTGAGGCCGCGGGG---------------GGGAACGGAGCAGAGGGCGGTCTGGACGTTGTTGGGGATC
 22 BhEt_9          57.5%  24.6%      TGGAGGACATCTTGAGGCCGCGGGG---------------AGGAACGGAGCAGAGGGCGGTCTGGACGTTGTTGGGGATC
 23 BhEt_8          59.9%  25.3%      TGGAGGACATCTTGAGGCCGCGGGG---------------AGGAACGGAGCAGAGGGCGGTCTGGACGTTGTTGGGGATC
 24 KaEt_6          82.7%  23.9%      TAGAGGACATAGTAAGTCCCCGAGG---------------AGGAATAGCGCAGAGAGCGGTCTGGATGTTGTTAGGGATC
 25 SaEt_5          91.0%  23.4%      TAGAGGACATAGTAAGTCCCCGAGG---------------AGGAATAGCGCAGAGAGCGGTCTGGATGTTGTTAGGGATC
 26 KaEt_7          82.2%  24.1%      TAGAGGACATAGTAAGTCCCCGAGG---------------AGGAATAGCGCAGAGAGCGGTCTGGATGTTGTTAGGGATC
 27 KaEt_4          82.4%  24.1%      TAGAGGACATAGTAAGTCCCCGAGG---------------AGGAATAGCGCAGAGAGCGGTCTGGATGTTGTTAGGGATC
 28 KaEt_9          77.2%  24.2%      TAGAGGACATAGTAAGTCCCCGAGG---------------AGGAATAGCGCAGAGAGCGGTCTGGATGTTGTTAGGGATC
 29 KaEt_8          81.9%  24.1%      TAGAGGACATAGTAAGTCCCCGAGG---------------AGGAATAGCGCAGAGAGCGGTCTGGATGTTGTTAGGGATC
 30 BeEt_7          81.8%  23.9%      TAGAGGACATAGTAAGTCCCCGAGG---------------AGGAATAGCGCAGAGAGCGGTCTGGATGTTGTTAGGGATC
 31 BeEt_3          82.1%  23.7%      TAGAGGACATAGTAAGTCCCCGAGG---------------AGGAATAGCGCAAAGAGCGGTCTGGATGTTGTTAGGGATC
 32 KhEt_8          82.2%  23.8%      TAGAGGACATAGTAAGTCCCCGAGG---------------AGGAATAGCGCAGAGAGCGGTCTGGATGTTGTTAGGGATC
 33 KhEt_7          78.5%  23.9%      TAGAGGACATAGTAAGTCCCCGAGG---------------AGGAATAGCGCAGAGAGCGGTCTGGATGTTGTTAGGGATC
 34 BhEt_2          61.8%  22.7%      TGGAGGACATCTTCAGGCCGCGGGG---------------AGGGATGGAGCAGAGGGCGGTCTGAACGTTGTTGGGGATC
 35 BhEt_1          52.4%  18.6%      ---------------------------------------------------------------TAACGTTGTTGGGGATC
 36 BeEt_4          83.5%  26.0%      TAGAGGACATCTTCAGGCCGCGGGG---------------AGGGATGGAGCAGAGGGCGGTCTGAACGTTGTTGGGGATC
 37 SaEt_3          81.9%  25.0%      TGGAGGACATCTTCAGGCCACGAGG---------------AGGAATGGAGCAGAGGGCGGTCTGGACATTGTTGGGGATC
 38 KhEt_9          82.7%  25.2%      TGGAGGACATCTTCAGGCCGCGAGG---------------AGGAATGGAGCAAAGAGCGGTCTGGACGTTGTTGGGGATC
 39 SaEt_4          82.7%  25.9%      TAGAGGACATCTTCAGGCCGCGGGG---------------AGGGATGGAGCAGAGGGCGGTCTGAACGTTGTTGGGGATC
 40 SaEt_8          91.0%  25.3%      TGGAGGACATCTTCAGGCCACGAGG---------------AGGAATGGAGCAGAGGGCAGTCTGGACATTGTTGGGGATC
 41 KaEt_5          91.7%  25.0%      TGGAGGACATCTTCAGGCCACGAGG---------------AGGAATGGAGCAGAGGGCAGTCTGGACATTGTTGGGGATC
 42 KaEt_2          82.2%  25.9%      TGGAGGACATCTTGAGGCCGCGAGG---------------AGGAATGGAGCAGAGGGCGGTCTGCACATTGTTGGGGATC
 43 KhEt_6          82.5%  25.9%      TGGAGGACATCTTGAGGCCGCGAGG---------------AGGAATGGAGCAGAGGGCGGTCTGCACATTGTTGGGGATC
 44 SaEt_2          70.8%  26.0%      TGGAGGACATCTTGAGGCCGCGAGG---------------AGGAATGGAGCAGAGGGCGGTCTGCACATTGTTGGGGATC
 45 SaEt_1          82.4%  25.9%      TGGAGGACATCTTGAGGCCGCGAGG---------------AGGAATGGAGCAGAGGGCGGTCTGCACATTGTTGGGGATC
    consensus/100%                    ..........................................................................ssssTC
```

```
    consensus/90%                   TsGAuGACATssTsAGsCCsCGuGG...............AGGuAsuGsuCAuAGuGCuGTCTGsAsuTTGTTuGGuATC
    consensus/80%                   TuGAuGACATssTuAGsCCsCGuGG...............AGGAAsuGsGCAGAGuGCGGTCTGGAsuTTGTTuGGuATC
    consensus/70%                   TuGAGGACATCTTuAGGCCsCGuGG...............AGGAAsGGsGCAGAGuGCGGTCTGGAsGTTGTTGGGGATC

                   cov    pid  241          :         .         .         .         .         3         .         . 320
  1 BhEt_3      100.0% 100.0%     ATCTCCAAGATCCGCGAGGAGTTCCCCGACCGCATGATGGCCACCT----------------------------------
  2 BeEt_2       60.7%  19.0%     CATTCAACGAAGTAAGAAGAGTTCTTGTTCTGGACGTTGCGCATCTGATCCTCGACCTCCTTCATAGCGACACGGCCACG
  3 BeEt_1       60.7%  19.7%     CATTCAACGAAGTAAGAAGAGTTCTTGTTCTGGACGTTGCGCATCTGATCCTCGACCTCCTTCATAGCGACACGGCCACG
  4 KhEt_1       82.5%  23.5%     CCCTCACCAAATTAGAAGAATTTCTGGTTCTGGACTTTGCGCACCTGGCCCTCACCCTCCTTCTGGGAGACCTTCCACGG
  5 BeEt_9       94.5%  24.6%     CACTCAACGAAGTAAGAGGAGTTCTTGTTCTGGACGTTGCGCATCTGGTCCTCGACCTCCTTCATGGCGACCTTACCACG
  6 SaEt_7       95.0%  23.9%     CACTCAACGAAGTAAGAGGAGTTCTTGTTCTGGACGTTGCGCATCTGGTCCTCGACCTCCTTCATGGCGACCTTACCACG
  7 SaEt_6       92.6%  24.3%     CACTCAACGAAGTAAGAGGAGTTCTTGTTCTGGACGTTGCGCATCTGGTCCTCGACCTCCTTCATGGCGACCTTACCACG
  8 SaEt_9       93.9%  23.7%     CACTCAACGAAGTAAGAGGAGTTCTTGTTCTGGACGTTGCGCATCTGGTCCTCGACCTCCTTCATGGCGACCTTACCACG
  9 BeEt_8       93.2%  22.9%     CACTCAACGAAGTAAGAGGAGTTCTTGTTCTGGACGTTGCGCATCTGGTCCTCGACCTCCTTCATGGCGACCTTACCACG
 10 BeEt_6       82.5%  22.4%     CACTCAACGAAGTAAGAGGAGTTCTTGTTCTGGACGTTGCGCATCTGGTCCTCGACCTCCTTCATGGCGACCTTACCACG
 11 BeEt_5       92.9%  24.2%     CACTCAACGAAGTAAGAGGAGTTCTTGTTCTGGACGTTGCGCATCTGGTCCTCGACCTCCTTCATGGCGACCTTACCACG
 12 BhEt_7       82.5%  24.1%     CACTCAACGAAGTAGGAAGAGTTCTTGTTCTGGACGTTGCGCATCTGGTCCTCGACCTCCTTCATGGAGACCTTACCACG
 13 BhEt_6       84.6%  23.2%     CACTCAACGAAGTAGGAAGAGTTCTTGTTCTGGACGTTGCGCATCTGGTCCTCGACCTCCTTCATGGAGACCTTACCACG
 14 BhEt_5       88.4%  24.2%     CACTCAACGAAGTAGGAAGAGTTCTTGTTCTGGACGTTGCGCATCTGGTCCTCGACCTCCTTCATGGAGACCTTACCACG
 15 BhEt_4       90.9%  24.9%     CACTCAACGAAGTAGGAAGAGTTCTTGTTCTGGACGTTGCGCATCTGGTCCTCGACCTCCTTCATGGAGACCTTACCACG
 16 KhEt_2       80.3%  24.2%     CACTCAACGAAGTAGGAGGAGTTCTTGTTCTGGACGTTGCGCATCTGGTCCTCAACCTCCTTCATGGAAACCTTACCACG
 17 KaEt_1       61.0%  24.9%     CACTCAACGAAGTACGAGGAGTTCTTGTTCTGGACGTTGCGCATCTGGTCCTCAACCTCCTTCATGGAGACCTTACCACG
 18 KhEt_5       81.4%  25.4%     CACTCAACGAAGTACGAGGAGTTCTTGTTCTGGACGTTGCGCATCTGGTCCTCAACCTCCTTCATGGAGACCTTACCACG
 19 KhEt_3       81.1%  24.6%     CACTCAACGAAGTACGAGGAGTTCTTGTTCTGGACGTTGCGCATCTGGTCCTCAACCTCCTTCATGGAGACCTTACCACG
 20 KhEt_4       82.5%  25.1%     CACTCAACGAAGTATGAGGAGTTCTTGTTCTGGACGTTGCGCATCTGGTCCTCAACCTCCTTCATGGAGACCTTACCACG
 21 KaEt_3       68.2%  24.9%     CACTCAACGAATTACGAGGAGTTCTTGTTCTGGACATTGCGCATCTGGTCCTCGACCTCCTTCATGGAGACCTTACCGCG
 22 BhEt_9       57.5%  24.6%     CACTCAACGAAGTACGAGGAGTTCTTGTTCTGGACGTTGCGCATCTGGTCCTCAACCTCCTTCATGGAGACCTTACCACG
 23 BhEt_8       59.9%  25.3%     CACTCAACGAAGTACGAGGAGTTCTTGTTCTGGACGTTGCGCATCTGGTCCTCAACCTCCTTCATGGAGACCTTACCACG
 24 KaEt_6       82.7%  23.9%     CACTCGACGAAGTAAGATGAGTTCTTGTTCTGAACGTTGCGCATCTGGTCCTCGACCTCCTTCATGGCGACGCGGCCACG
 25 SaEt_5       91.0%  23.4%     CACTCGACGAAGTAAGATGAGTTCTTGTTCTGAACGTTGCGCATCTGGTCCTCGACCTCCTTCATGGCGACGCGGCCACG
 26 KaEt_7       82.2%  24.1%     CACTCGACGAAGTAAGATGAGTTCTTGTTCTGAACGTTGCGCATCTGGTCCTCGACCTCCTTCATGGCGACGCGGCCACG
 27 KaEt_4       82.4%  24.1%     CACTCGACGAAGTAAGATGAGTTCTTGTTCTGAACGTTGCGCATCTGGTCCTCGACCTCCTTCATGGCGACGCGGCCACG
 28 KaEt_9       77.2%  24.2%     CACTCGACGAAGTAAGATGAGTTCTTGTTCTGAACGTTGCGCATCTGGTCCTCGACCTCCTTCATGGCGACGCGGCCACG
 29 KaEt_8       81.9%  24.1%     CACTCGACGAAGTAAGATGAGTTCTTGTTCTGAACGTTGCGCATCTGGTCCTCGACCTCCTTCATGGCGACGCGGCCACG
 30 BeEt_7       81.8%  23.9%     CACTCGACGAAGTAAGATGAGTTCTTGTTCTGAACGTTGCGCATCTGGTCCTCGACCTCCTTCATGGCGACGCGGCCACG
 31 BeEt_3       82.1%  23.7%     CACTCGACGAAGTAAGATGAGTTCTTGTTCTGAACGTTGCGCATCTGGTCCTCGACCTCCTTCATGGCGACGCGGCCACG
 32 KhEt_8       82.2%  23.8%     CACTCGACGAAGTAAGATGAGTTCTTGTTCTGAACGTTGCGCATCTGGTCCTCGACCTCCTTCATGGCGACGCGGCCACG
 33 KhEt_7       78.5%  23.9%     CACTCGACGAAGTAAGATGAGTTCTTGTTCTGAACGTTGCGCATCTGGTCCTCGACCTCCTTCATGGCGACGCGGCCACG
 34 BhEt_2       61.8%  22.7%     CACTCAACGAAGTATGAGGAGTTCTTGTTCTGGACGTTGCGCATCTGGTCCTCGACCTCCTTCATGGAGACCTTACCACG
 35 BhEt_1       52.4%  18.6%     CACTCAACGAAGTATGAGGAGTTCTTGTTCTGGACGTTGCGCATCTGGTCCTCGACCTCCTTCATGGAGACCTTACCACG
 36 BeEt_4       83.5%  26.0%     CACTCAACGAAGTATGAGGAGTTCTTGTTCTGGACGTTGCGCATCTGGTCCTCGACCTCCTTCATGGAGACCTTACCACG
 37 SaEt_3       81.9%  25.0%     CACTCAACAAAGTAGGAAGAGTTCTTGTTCTGGACGTTGCGCATCTGGTCCTCAACCTCCTTCATGGAGACCTTACCGCG
 38 KhEt_9       82.7%  25.2%     CACTCAACGAAGTAGGACGAGTTCTTGTTCTGGACGTTGCGCATCTGGTCCTCAACCTCCTTCATGGAGACCTTGCCACG
 39 SaEt_4       82.7%  25.9%     CACTCAACGAAGTATGAGGAGTTCTTGTTCTGGACGTTGCGCATCTGGTCCTCGACCTCCTTCATGGAGACCTTACCACG
 40 SaEt_8       91.0%  25.3%     CACTCAACAAAGTAGGAAGAGTTCTTGTTCTGGACGTTGCGCATCTGGTCCTCAACCTCCTTCATGGAGACCTTACCGCG
 41 KaEt_5       91.7%  25.0%     CACTCAACAAAGTAGGAAGAGTTCTTGTTCTGGACGTTGCGCATCTGGTCCTCAACCTCCTTCATGGAGACCTTACCGCG
 42 KaEt_2       82.2%  25.9%     CACTCAACGAAGTAGGAAGAGTTCTTGTTCTGGACGTTGCGCATCTGGTCCTCAACCTCCTTCATCGAGACCTTACCGCG
 43 KhEt_6       82.5%  25.9%     CACTCAACGAAGTAGGAAGAGTTCTTGTTCTGGACGTTGCGCATCTGGTCCTCAACCTCCTTCATCGAGACCTTACCGCG
 44 SaEt_2       70.8%  26.0%     CACTCAACGAAGTAGGAAGAGTTCTTGTTCTGGACGTTGCGCATCTGGTCCTCAACCTCCTTCATCGAGACCTTACCGCG
 45 SaEt_1       82.4%  25.9%     CACTCAACGAAGTAGGAAGAGTTCTTGTTCTGGACGTTGCGCATCTGGTCCTCAACCTCCTTCATCGAGACCTTACCGCG
    consensus/100%                  sssTCsssuAsssusuAsuAsTTCsssssCsGsAsssTGssCAsCT..................................
    consensus/90%                   CACTCuACGAAGTAsGAsGAGTTCTTGTTCTGuACGTTGCGCATCTGGTCCTCuACCTCCTTCATsGsGACsssuCCuCG
    consensus/80%                   CACTCuACGAAGTAsGAsGAGTTCTTGTTCTGuACGTTGCGCATCTGGTCCTCuACCTCCTTCATGGsGACsssuCCuCG
    consensus/70%                   CACTCAACGAAGTAuGAuGAGTTCTTGTTCTGGACGTTGCGCATCTGGTCCTCuACCTCCTTCATGGsGACCTTuCCACG

                   cov    pid  321          .         .         :         .         .         .         .       4 400
  1 BhEt_3      100.0% 100.0%     --------------------------------------------------------------------------------
  2 BeEt_2       60.7%  19.0%     GCTGTTCAAAGTCAGTAAATAATTTCC--------ATGCGCAAATCGGGTTCACTCACAAAATGGCCGAGCAGGTCAGGT
  3 BeEt_1       60.7%  19.7%     GCTGTTCAAAGTCAGTAAATAATTTCC--------ATGCGCAAATCGGGTTCACTCACAAAATGGCCGAGCAGGTCAGGT
  4 KhEt_1       82.5%  23.5%     AAG----------------------------------------------------------TGGGCAGAGCGGGTCAGGT
  5 BeEt_9       94.5%  24.6%     GCTATTGAACATAATTAGCATTGAGACTCAACCAGATTTTATCTGTCATATGACTTACAAGATGGCACAGCAGGTCAGGT
  6 SaEt_7       95.0%  23.9%     GCTATTGAACATAATTAGCATTGAGACTCAACCAGATTTTATCTGTCATATGACTTACAAGATGGCACAGCAGGTCAGGT
  7 SaEt_6       92.6%  24.3%     GCTATTGAACATAATTAGCATTGAGACTCAACCAGATTTTATCTGTCATATGACTTACAAGATGGCACAGCAGGTCAGGT
  8 SaEt_9       93.9%  23.7%     GCTATTGAACATAATTAGCATTGAGACTCAACCAGATTTTATCTGTCATATGACTTACAAGATGGCACAGCAGGTCAGGT
  9 BeEt_8       93.2%  22.9%     GCTATTGAACATAATTAGCATTGAGACTCAACCAGATTTTATCTGTCATATGACTTACAAGATGGCACAGCAGGTCAGGT
 10 BeEt_6       82.5%  22.4%     GCTATTGAACATAATTAGCATTGAGACTCAACCAGATTTTATCTGTCATATGACTTACAAGATGGCACAGCAGGTCAGGT
 11 BeEt_5       92.9%  24.2%     GCTATTGAACATAATTAGCATTGAGACTCAACCAGATTTTATCTGTCATATGACTTACAAGATGGCACAGCAGGTCAGGT
 12 BhEt_7       82.5%  24.1%     GAA---------------------------------------------------------GTAGGCAGAGCAAGTCAGGT
 13 BhEt_6       84.6%  23.2%     GAA---------------------------------------------------------GTAGGCAGAGCAAGTCAGGT
 14 BhEt_5       88.4%  24.2%     GAA---------------------------------------------------------GTAGGCAGAGCAAGTCAGGT
 15 BhEt_4       90.9%  24.9%     GAA---------------------------------------------------------GTAGGCAGAGCAAGTCAGGT
 16 KhEt_2       80.3%  24.2%     GAA---------------------------------------------------------GTAGGCAGAGCAGGTGAGGT
 17 KaEt_1       61.0%  24.9%     GAA---------------------------------------------------------GTAGGCAGAGCAGGTCAGGT
 18 KhEt_5       81.4%  25.4%     GAA---------------------------------------------------------GTAGGCAGAGCAGGTCAGGT
 19 KhEt_3       81.1%  24.6%     GAA---------------------------------------------------------GTAGGCAGAGCAGGTCAGGT
 20 KhEt_4       82.5%  25.1%     GAA---------------------------------------------------------GTAGGCAGAGCAGGTCAGGT
 21 KaEt_3       68.2%  24.9%     GAA---------------------------------------------------------GTAGGCCGAGCAGGTCAGGT
 22 BhEt_9       57.5%  24.6%     GAA---------------------------------------------------------GTAGGCAGAGCAGGTCAGGT
```

```
23 BhEt_8        59.9%  25.3%     GAA---------------------------------------------------------GTAGGCAGAGCAGGTCAGGT
24 KaEt_6        82.7%  23.9%     GAA---------------------------------------------------------GATGGCTTAACAGGTCAGGT
25 SaEt_5        91.0%  23.4%     GAA---------------------------------------------------------GATGGCAGAACAGGTCAGGT
26 KaEt_7        82.2%  24.1%     GAA---------------------------------------------------------GATGGCAGAACAGGTCAGGT
27 KaEt_4        82.4%  24.1%     GAA---------------------------------------------------------GATGGCAGAACAGGTCAGGT
28 KaEt_9        77.2%  24.2%     GAA---------------------------------------------------------GATGGCAGAACAGGTCAGGT
29 KaEt_8        81.9%  24.1%     GAA---------------------------------------------------------GATGGCAGAACAGGTCAGGT
30 BeEt_7        81.8%  23.9%     GAA---------------------------------------------------------GATGGCAGAACAGGTCAGGT
31 BeEt_3        82.1%  23.7%     GAA---------------------------------------------------------GATGGCAGAACAGGTCAGGT
32 KhEt_8        82.2%  23.8%     GAA---------------------------------------------------------GATGGCAGAACAGGTCAGGT
33 KhEt_7        78.5%  23.9%     GAA---------------------------------------------------------GATGGCAGAACAGGTCAGGT
34 BhEt_2        61.8%  22.7%     GAA---------------------------------------------------------GTAGGCAGAGCAGGTCAGGT
35 BhEt_1        52.4%  18.6%     GAA---------------------------------------------------------GTAGGCAGAGCAGGTCAGGT
36 BeEt_4        83.5%  26.0%     GAA---------------------------------------------------------GTAGGCAGAGCAGGTCAGGT
37 SaEt_3        81.9%  25.0%     GAA---------------------------------------------------------GTAGGCGGAGCAGGTCAGGT
38 KhEt_9        82.7%  25.2%     GAA---------------------------------------------------------GTAGGCAGAGCAGGTCAGGT
39 SaEt_4        82.7%  25.9%     GAA---------------------------------------------------------GTAGGCAGAGCAGGTCAGGT
40 SaEt_8        91.0%  25.3%     GAA---------------------------------------------------------GTAGGCGGAGCAGGTCAGGT
41 KaEt_5        91.7%  25.0%     GAA---------------------------------------------------------GTAGGCGGAGCAGGTCAGGT
42 KaEt_2        82.2%  25.9%     GAA---------------------------------------------------------GTAGGCGGAGCAGGTCAGGT
43 KhEt_6        82.5%  25.9%     GAA---------------------------------------------------------GTAGGCGGAGCAGGTCAGGT
44 SaEt_2        70.8%  26.0%     GAA---------------------------------------------------------GTAGGCGGAGCAGGTCAGGT
45 SaEt_1        82.4%  25.9%     GAA---------------------------------------------------------GTAGGCGGAGCAGGTCAGGT
   consensus/100%                 ................................................................................
   consensus/90%                  Gss.........................................................GssGGCssAuCAuGTCAGGT
   consensus/80%                  Gss.........................................................GssGGCuGAuCAGGTCAGGT
   consensus/70%                  GAA.........................................................GssGGCAGAGCAGGTCAGGT

                 cov    pid   401         .         .         .         .         :         .         .         . 480
 1 BhEt_3       100.0% 100.0%     ---------------------------------------------------TCTCCGTTGTGCCCTCCCCCAAGGTCT--
 2 BeEt_2        60.7%  19.0%     AGCGACCATTGCGGAAGTCCGAAGCAGCCATCATGTTCTTGGGGTCGAACATCTGTTGGGTCAACTCAGGAACGCTGACA
 3 BeEt_1        60.7%  19.7%     AGCGACCATTGCGGAAGTCCGAAGCAGCCATCATGTTCTTGGGGTCGAACATCTGTTGGGTCAACTCAGGAACGCTGACA
 4 KhEt_1        82.5%  23.5%     AACGACCGTTGCGGAA-TTCGAAACAGCCTTCAGTTTCTGGGGGTCAAACATCTGCTGGGTGACCTCGGAAACCTTGACG
 5 BeEt_9        94.5%  24.6%     AGCGACCGTTGCGGAAGTCAGAAGCAGCCATCATGTTCTTGGGGTCGAACATCTGCTGGGTCAGCTCAGGAACGCTGACG
 6 SaEt_7        95.0%  23.9%     AGCGACCGTTGCGGAAGTCAGAAGCAGCCATCATGTTCTTGGGGTCGAACATCTGCTGGGTCAGCTCAGGAACGCTGACG
 7 SaEt_6        92.6%  24.3%     AGCGACCGTTGCGGAAGTCAGAAGCAGCCATCATGTTCTTGGGGTCGAACATCTGCTGGGTCAGCTCAGGAACGCTGACG
 8 SaEt_9        93.9%  23.7%     AGCGACCGTTGCGGAAGTCAGAAGCAGCCATCATGTTCTTGGGGTCGAACATCTGCTGGGTCAGCTCAGGAACGCTGACG
 9 BeEt_8        93.2%  22.9%     AGCGACCGTTGCGGAAGTCAGAAGCAGCCATCATGTTCTTGGGGTCGAACATCTGCTGGGTCAGCTCAGGAACGCTGACG
10 BeEt_6        82.5%  22.4%     AGCGACCGTTGCGGAAGTCAGAAGCAGCCATCATGTTCTTGGGGTCGAACATCTGCTGGGTCAGCTCAGGAACGCTGACG
11 BeEt_5        92.9%  24.2%     AGCGACCGTTGCGGAAGTCAGAAGCAGCCATCATGTTCTTGGGGTCGAACATCTGCTGGGTCAGCTCAGGAACGCTGACG
12 BhEt_7        82.5%  24.1%     AACGACCGTTGCGGAAGTCAGAAGCAGCCATCATGTTCTTGGGGTCGAACATCTGCTGGGTGAGCTCGGGGACGGTGACG
13 BhEt_6        84.6%  23.2%     AACGACCGTTGCGGAAGTCAGAAGCAGCCATCATGTTCTTGGGGTCGAACATCTGCTGGGTGAGCTCGGGGACGGTGACG
14 BhEt_5        88.4%  24.2%     AACGACCGTTGCGGAAGTCAGAAGCAGCCATCATGTTCTTGGGGTCGAACATCTGCTGGGTGAGCTCGGGGACGGTGACG
15 BhEt_4        90.9%  24.9%     AACGACCGTTGCGGAAGTCAGAAGCAGCCATCATGTTCTTGGGGTCGAACATCTGCTGGGTGAGCTCGGGGACGGTGACG
16 KhEt_2        80.3%  24.2%     AACGACCGTTGCGGAAGTCAGAAGCAGCCATCATGTTCTTGGGGTCGAACATCTGCTGGGTGAGCTCGGGAACGGTGACG
17 KaEt_1        61.0%  24.9%     AACGACCGTTGCGGAAGTCGGAAGCAGCCATCATGTTCTTGGGGTCGAACATCTGCTGGGTGAGCTCGGGAACAGTGACG
18 KhEt_5        81.4%  25.4%     AACGACCGTTGCGGAAGTCGGAAGCAGCCATCATGTTCTTGGGGTCGAACATCTGCTGGGTGAGCTCGGGAACAGTGACG
19 KhEt_3        81.1%  24.6%     AACGACCGTTGCGGAAGTCGGAAGCAGCCATCATGTTCTTGGGGTCGAACATCTGCTGGGTGAGCTCGGGAACAGTGACG
20 KhEt_4        82.5%  25.1%     AACGACCGTTGCGGAAGTCGGAAGCAGCCATCATGTTCTTGGGGTCGAACATCTGCTGGGTGAGCTCGGGAACAGTGACG
21 KaEt_3        68.2%  24.9%     AACGACCGTTGCGGAAGTCAGAAGCAGCCATCATGTTCTTGGGGTCGAACATCTGCTGGGTGAGCTCGGGAACAGTGACG
22 BhEt_9        57.5%  24.6%     AACGACCGTTGCGGAAGTCGGAAGCAGCCATCATGTTCTTGGGGTCGAACATCTGCTGGGTGAGCTCGGGAACA------
23 BhEt_8        59.9%  25.3%     AACGACCGTTGCGGAAGTCGGAAGCAGCCATCATGTTCTTGGGGTCGAACATCTGCTGGGTGAGCTCGGGAACAGTGACG
24 KaEt_6        82.7%  23.9%     AACGACCGTTGCGGAAGTCGGAAGCAGCCATCATGTTCTTGGGGTCGAACATCTGCTGGGTCAGCTCAGGAACGCTGACA
25 SaEt_5        91.0%  23.4%     AACGACCGTTGCGGAAGTCGGAAGCAGCCATCATGTTCTTGGGGTCGAACATCTGCTGGGTCAGCTCAGGAACGCTGACA
26 KaEt_7        82.2%  24.1%     AACGACCGTTGCGGAAGTCGGAAGCAGCCATCATGTTCTTGGGGTCGAACATCTGCTGGGTCAGCTCAGGAACGCTGACA
27 KaEt_4        82.4%  24.1%     AACGACCGTTGCGGAAGTCGGAAGCAGCCATCATGTTCTTGGGGTCGAACATCTGCTGGGTCAGCTCAGGAACGCTGACA
28 KaEt_9        77.2%  24.2%     AACGACCGTTGCGGAAGTCGGAAGCAGCCATCATGTTCTTGGGGTCGAACATCTGCTGGGTCAGCTCAGGAACGCTGACA
29 KaEt_8        81.9%  24.1%     AACGACCGTTGCGGAAGTCGGAAGCAGCCATCATGTTCTTGGGGTCGAACATCTGCTGGGTCAGCTCAGGAACGCTGACA
30 BeEt_7        81.8%  23.9%     AACGACCGTTGCGGAAGTCGGAAGCAGCCATCATGTTCTTGGGGTCGAACATCTGCTGGGTCAGCTCAGGAACGCTGACA
31 BeEt_3        82.1%  23.7%     AACGACCGTTGCGGAAGTCGGAAGCAGCCATCATGTTCTTGGGGTCGAACATCTGCTGGGTCAGCTCAGGAACGCTGACA
32 KhEt_8        82.2%  23.8%     AACGACCGTTGCGGAAGTCGGAAGCAGCCATCATGTTCTTGGGGTCGAACATCTGCTGGGTCAGCTCAGGAACGCTGACA
33 KhEt_7        78.5%  23.9%     AACGACCGTTGCGGAAGTCGGAAGCAGCCATCATGTTCTTGGGGTCGAACATCTGCTGGGTCAGCTCAGGAACGCTGACA
34 BhEt_2        61.8%  22.7%     AGCGACCGTTGCGGAAGTCGGAAGCAGCCATCATGTTCTTGGGGTCGAACATCTGCTGGGTGAGCTCGGGAACGGTGACG
35 BhEt_1        52.4%  18.6%     AGCGACCGTTGCGGAAGTCGGAAGCAGCCATCATGTTCTTGGGGTCGAACATCTGCTGGGTGAGCTCGGGAACGGTGACG
36 BeEt_4        83.5%  26.0%     AGCGACCGTTGCGGAAGTCGGAAGCAGCCATCATGTTCTTGGGGTCGAACATCTGCTGGGTGAGCTCGGGAACGGTGACG
37 SaEt_3        81.9%  25.0%     AGCGACCGTTGCGGAAGTCGGAGGCGGCCATCATGTTCTTGGGATCGAACATTTGCTGGGTGAGCTCGGGAACGGTGACG
38 KhEt_9        82.7%  25.2%     AGCGACCGTTGCGGAAGTCGGAGGCAGCCATCATGTTCTTGGGGTCGAACATTTGCTGGGTGAGCTCGGGAACGGTGACG
39 SaEt_4        82.7%  25.9%     AGCGACCGTTGCGGAAGTCGGAAGCAGCCATCATGTTCTTGGGGTCGAACATCTGCTGGGTGAGCTCGGGAACGGTGACG
40 SaEt_8        91.0%  25.3%     AGCGACCGTTGCGGAAGTCGGAGGCGGCCATCATGTTCTTGGGGTCGAACATTTGCTGGGTGAGCTCGGGAACGGTGACG
41 KaEt_5        91.7%  25.0%     AGCGACCGTTGCGGAAGTCGGAGGCGGCCATCATGTTCTTGGGGTCGAACATTTGCTGGGTGAGCTCGGGAACGGTGACG
42 KaEt_2        82.2%  25.9%     AGCGACCGTTGCGGAAGTCGGAAGCAGCCATCATGTTCTTGGGGTCGAACATTTGCTGGGTGAGCTCGGGAACGGTGACG
43 KhEt_6        82.5%  25.9%     AGCGACCGTTGCGGAAGTCGGAAGCAGCCATCATGTTCTTGGGGTCGAACATTTGCTGGGTGAGCTCGGGAACGGTGACG
44 SaEt_2        70.8%  26.0%     AGCGACCGTTGCGGAAGTCGGAAGCAGCCATCATGTTCTTGGGGTCGAACATTTGCTGGGTGAGCTCGGGAACGGTGACG
45 SaEt_1        82.4%  25.9%     AGCGACCGTTGCGGAAGTCGGAAGCAGCCATCATGTTCTTGGGGTCGAACATTTGCTGGGTGAGCTCGGGAACGGTGACG
   consensus/100%                 ...................................................TsTsssssGTsssCTCssssAss......
   consensus/90%                  AuCGACCGTTGCGGAAGTCuGAuGCAGCCATCATGTTCTTGGGGTCGAACATsTGCTGGGTsAGCTCuGGuACusTGACu
   consensus/80%                  AuCGACCGTTGCGGAAGTCuGAAGCAGCCATCATGTTCTTGGGGTCGAACATCTGCTGGGTsAGCTCuGGAACGsTGACu
   consensus/70%                  AuCGACCGTTGCGGAAGTCuGAAGCAGCCATCATGTTCTTGGGGTCGAACATCTGCTGGGTsAGCTCuGGAACGsTGACu
```

```
                    cov    pid  481         .         5         .         .         .         :         . 560
  1 BhEt_3         100.0% 100.0%    ---------------CCGACACCGTTGTCGAG------------------------------------------------
  2 BeEt_2          60.7%  19.0%    GCGCGGAAAGAGTGAGCACCACGGCTGGTCAGAGGAGCAAATCCAACCATGAATAAGTGTAGACTAGGGAAAGGCACCAT
  3 BeEt_1          60.7%  19.7%    GCGCGGAAAGAGTGAGCACCACGGCTGGTCAGAGGAGCAAATCCAACCATGAATAAGTGTAGACTAGGGAAAGGCACCAT
  4 KhEt_1          82.5%  23.5%    GCCG-GAAGGATTGGGCCCCCCGGCGGGCCAGAGAACCAAACCCAACCTGAAAAAATTGAAAACAGGGAAAGGAACCCTG
  5 BeEt_9          94.5%  24.6%    GCGCGGAAAGAGTGGGCACCACGGCTGGTCAGGGGAGCGAATCCGACCATGAAGAAGTGCAGGCGAGGGAAAGGAACCAT
  6 SaEt_7          95.0%  23.9%    GCGCGGAAAGAGTGGGCACCACGGCTGGTCAGGGGAGCGAATCCGACCATGAAGAAGTGCAGGCGAGGGAAAGGAACCAT
  7 SaEt_6          92.6%  24.3%    GCGCGGAAAGAGTGGGCACCACGGCTGGTCAGGGGAGCGAATCCGACCATGAAGAAGTGCAGGCGAGGGAAAGGAACCAT
  8 SaEt_9          93.9%  23.7%    GCGCGGAAAGAGTGGGCACCACGGCTGGTCAGGGGAGCGAATCCGACCATGAAGAAGTGCAGGCGAGGGAAAGGAACCAT
  9 BeEt_8          93.2%  22.9%    GCGCGGAAAGAGTGGGCACCACGGCTGGTCAGGGGAGCGAATCCGACCATGAAGAAGTGCAGGCGAGGGAAAGGAACCAT
 10 BeEt_6          82.5%  22.4%    GCGCGGAAAGAGTGGGCACCACGGCTGGTCAGGGGAGCGAATCCGACCATGAAGAAGTGCAGGCGAGGGAAAGGAACCAT
 11 BeEt_5          92.9%  24.2%    GCGCGGAAAGAGTGGGCACCACGGCTGGTCAGGGGAGCGAATCCGACCATGAAGAAGTGCAGGCGAGGGAAAGGAACCAT
 12 BhEt_7          82.5%  24.1%    GCGCGGAAGGAGTGGGCGCCACGGCTGGTCAGAGGAGCGAAACCGACCATGAAGAAGTGGAGACGGGGGAA---------
 13 BhEt_6          84.6%  23.2%    GCGCGGAAGGAGTGGGCGCCACGGCTGGTCAGAGGAGCGAAACCGACCATGAAGAAGTGGAGACGGGGGAAGGGAACCAT
 14 BhEt_5          88.4%  24.2%    GCGCGGAAGGAGTGGGCGCCACGGCTGGTCAGAGGAGCGAAACCGACCATGAAGAAGTGGAGACGGGGGAAGGGAACCAT
 15 BhEt_4          90.9%  24.9%    GCGCGGAAGGAGTGGGCGCCACGGCTGGTCAGAGGAGCGAAACCGACCATGAAGAAGTGGAGACGGGGGAAGGGAACCAT
 16 KhEt_2          80.3%  24.2%    GCGCGGAAAGAGTGGGCGCCACGGCTGGTCAGAGGAGCGAAACCGACCATGAAGAAGTGGAGACGGGGGAAGGGAACCAT
 17 KaEt_1          61.0%  24.9%    GCACGGAAGGAGTGGGCGCCACGGCTGGTCAGAGGAGCGAAACCGACCATGAAGAAGTGGAGACGGGGGAAGGGAACCAT
 18 KhEt_5          81.4%  25.4%    GCACGGAAGGAGTGGGCGCCACGGCTGGTCAGAGGAGCGAAACCGACCATGAAGAAGTGGAGACGGGGGAAGGGAACCAT
 19 KhEt_3          81.1%  24.6%    GCACGGAAGGAGTGGGCGCCACGGCTGGTCAGAGGAGCGAAACCGACCATGAAGAAGTGGAGACGGGGGAAGGGAACCAT
 20 KhEt_4          82.5%  25.1%    GCACGGAAGGAGTGGGCGCCACGGCTGGTCAGAGGAGCGAAACCGACCATGAAGAAGTGGAGACGGGGGAAGGGAACCAT
 21 KaEt_3          68.2%  24.9%    GCACGGAAGGAGTGGGCGCCACGGCTGGTCAGAGGAGCGAAACCGACCATGAAGAAGTGGAGACGGGGGAAGGGAACCAT
 22 BhEt_9          57.5%  24.6%    --------------------------GGTCAGAGGAGCGAAACCGACCATGAAGAAGTGGAGACGGGGGAAGGGAACCAT
 23 BhEt_8          59.9%  25.3%    GCACGGAAGGAGTGGGCGCCACGGCTGGTCAGAGGAGCGAAACCGACCATGAAGAAGTGGAGACGGGGGAAGGGAACCAT
 24 KaEt_6          82.7%  23.9%    GCGCGGAAAGAGTGAGCACCACGGCTGGTCAGGGGAGCGAAGCCGACCATGAAGAAGTGGAGACGAGGGAAAGGAACCAT
 25 SaEt_5          91.0%  23.4%    GCGCGGAAAGAGTGAGCACCACGGCTGGTCAGGGGAGCGAAGCCGACCATGAAGAAGTGGAGACGAGGGAAAGGAACCAT
 26 KaEt_7          82.2%  24.1%    GCGCGGAAAGAGTGAGCACCACGGCTGGTCAGGGGAGCGAAGCCGACCATGAAGAAGTGGAGACGAGGGAAAGGAACCAT
 27 KaEt_4          82.4%  24.1%    GCGCGGAAAGAGTGAGCACCACGGCTGGTCAGGGGAGCGAAGCCGACCATGAAGAAGTGGAGACGAGGGAAAGGAACCAT
 28 KaEt_9          77.2%  24.2%    GCGCGGAAAGAGTGAGCACCACGGCTGGTCAGGGGAGCGAAGCCGACCATGAAGAAGTGGAGACGAGGGAAAGGAACCAT
 29 KaEt_8          81.9%  24.1%    GCGCGGAAAGAGTGAGCACCACGGCTGGTCAGGGGAGCGAAGCCGACCATGAAGAAGTGGAGACGAGGGAAAGGAACCAT
 30 BeEt_7          81.8%  23.9%    GCGCGGAAAGAGTGAGCACCACGGCTGGTCAGGGGAGCGAAGCCGACCATGAAGAAGTGGAGACGAGGGAAAGGAACCAT
 31 BeEt_3          82.1%  23.7%    GCGCGGAAAGAGTGAGCACCACGGCTGGTCAGGGGAGCGAAGCCGACCATGAAGAAGTGGAGACGAGGGAAAGGAACCAT
 32 KhEt_8          82.2%  23.8%    GCGCGGAAAGAGTGAGCACCACGGCTGGTCAGGGGAGCGAAGCCGACCATGAAGAAGTGGAGACGAGGGAAAGGAACCAT
 33 KhEt_7          78.5%  23.9%    GCGCGGAAAGAGTGAGCACCACGGCTGGTCAGGGGAGCGAAGCCGACCATGAAGAAGTGGAGACGAGGGAAAGGAACCAT
 34 BhEt_2          61.8%  22.7%    GCGCGGAAGGAGTGGGCACCGCGGCTGGTGAGGGGAGCGAATCCGACCATGAAGAAGTGGAGACGGGGGAAGGGAACCAT
 35 BhEt_1          52.4%  18.6%    GCGCGGAAGGAGTGGGCACCGCGGCTGGTGAGGGGAGCGAATCCGACCATGAAGAAGTGGAGACGGGGGAAGGGAACCAT
 36 BeEt_4          83.5%  26.0%    GCGCGGAAGGAGTGGGCACCGCGGCTGGTGAGGGGAGCGAATCCGACCATGAAGAAGTGGAGACGGGGGAAGGGAACCAT
 37 SaEt_3          81.9%  25.0%    GCGCGGAAGGAGTTGGCGCCACGGCTGGTAAGAGGAGCGAAACCAACCATGAAGAAGTGGAGACGGGGGAAGGGAACCAT
 38 KhEt_9          82.7%  25.2%    GCGCGGAAGGAGTGGGCGCCACGGCTGGTAAGAGGAGCGAAACCAACCATGAAGAAGTGGAGACGGGGGAAGGGAACCAT
 39 SaEt_4          82.7%  25.9%    GCGCGGAAGGAGTGGGCACCGCGGCTGGTGAGGGGAGCGAATCCGACCATGAAGAAGTGGAGACGGGGGAAGGGAACCAT
 40 SaEt_8          91.0%  25.3%    GCGCGGAAGGAGTTGGCGCCACGGCTGGTAAGAGGAGCGAAACCAACCATGAAGAAGTGGAGACGGGGGAAAGGAACCAT
 41 KaEt_5          91.7%  25.0%    GCGCGGAAGGAGTTGGCGCCACGGCTGGTAAGAGGAGCGAAACCAACCATGAAGAAGTGGAGACGGGGGAAAGGAACCAT
 42 KaEt_2          82.2%  25.9%    GCGCGGAAGGAGTGGGCGCCACGGCTGGTGAGAGGAGCGAAACCAACCATGAAGAAGTGGAGACGAGGGAAGGGAACCAT
 43 KhEt_6          82.5%  25.9%    GCGCGGAAGGAGTGGGCGCCACGGCTGGTGAGAGGAGCGAAACCAACCATGAAGAAGTGGAGACGAGGGAAGGGAACCAT
 44 SaEt_2          70.8%  26.0%    GCGCGGAAGGAGTGGGCGCCACGGCTGGTGAGAGGAGCGAAACCAACCATGAAGAAGTGGAGACGAGGGAAGGGAACCAT
 45 SaEt_1          82.4%  25.9%    GCGCGGAAGGAGTGGGCGCCACGGCTGGTGAGAGGAGCGAAACCAACCATGAAGAAGTGGAGACGAGGGAAGGGAACCAT
    consensus/100%                  ..........................GsssAG................................................
    consensus/90%                   GCuCGGAAuGAGTsuGCuCCuCGGCTGGTsAGuGGAGCGAAsCCuACCATGAAGAAGTGsAGuCGuGGGAAuGGAACCAT
    consensus/80%                   GCGCGGAAuGAGTGuGCuCCACGGCTGGTsAGuGGAGCGAAsCCuACCATGAAGAAGTGsAGACGuGGGAAuGGAACCAT
    consensus/70%                   GCGCGGAAuGAGTGuGCuCCACGGCTGGTCAGuGGAGCGAAsCCGACCATGAAGAAGTGGAGACGuGGGAAuGGAACCAT

                    cov    pid  561         .         .         .         6         .         .         . 640
  1 BhEt_3         100.0% 100.0%    ---------------------------------------------------------CCCTACAACGCCACTCTCTCCAT
  2 BeEt_2          60.7%  19.0%    GTTGACGGCGAGCTTTCGGAGATCGGAGTTCAGCTGACCGGGGAAACGGATACAGGTGGTGACACCGGACATAACATCAG
  3 BeEt_1          60.7%  19.7%    GTTGACGGCGAGCTTTCGGAGATCGGAGTTCAGCTGACCGGGGAAACGGATACAGGTGGTGACACCGGACATAACATCAG
  4 KhEt_1          82.5%  23.5%    GTTAACGGCAGCTTTCCGAGGGTCAAATTTCACTTGACCGGGAAACCGAGACCGGGTGGTGACCCCCGACATGACGGCGA
  5 BeEt_9          94.5%  24.6%    GTTGACGGCCAGCTTGCGCAGGTCAGAGTTCAGCTGACCAGGGAAACGCAGGCAGGTGGTAACACCGGACATGACGGCGG
  6 SaEt_7          95.0%  23.9%    GTTGACGGCCAGCTTGCGCAGGTCAGAGTTCAGCTGACCAGGGAAACGCAGGCAGGTGGTAACACCGGACATGACGGCGG
  7 SaEt_6          92.6%  24.3%    GTTGACGGCCAGCTTGCGCAGGTCAGAGTTCAGCTGACCAGGGAAACGCAGGCAGGTGGTAACACCGGACATGACGGCGG
  8 SaEt_9          93.9%  23.7%    GTTGACGGCCAGCTTGCGCAGGTCAGAGTTCAGCTGACCAGGGAAACGCAGGCAGGTGGTAACACCGGACATGACGGCGG
  9 BeEt_8          93.2%  22.9%    GTTGACGGCCAGCTTGCGCAGGTCAGAGTTCAGCTGACCAGGGAAACGCAGGCAGGTGGTAACACCGGACATGACGGCGG
 10 BeEt_6          82.5%  22.4%    GTTGACGGCCAGCTTGCGCAGGTCAGAGTTCAGCTGACCAGGGAAACGCAGGCAGGTGGTAACACCGGACATGACGGCGG
 11 BeEt_5          92.9%  24.2%    GTTGACGGCCAGCTTGCGCAGGTCAGAGTTCAGCTGACCAGGGAAACGCAGGCAGGTGGTAACACCGGACATGACGGCGG
 12 BhEt_7          82.5%  24.1%    --------------------------------------------------------------------------------
 13 BhEt_6          84.6%  23.2%    GTTGACGGCCAACTTCCTCAGGTCAGAGTTCAACTGACCGGGGAAACGCAGGCAGGTGGTGACACCCGACATGACGGCGG
 14 BhEt_5          88.4%  24.2%    GTTGACGGCCAACTTCCTCAGGTCAGAGTTCAACTGACCGGGGAAACGCAGGCAGGTGGTGACACCCGACATGACGGCGG
 15 BhEt_4          90.9%  24.9%    GTTGACGGCCAACTTCCTCAGGTCAGAGTTCAACTGACCGGGGAAACGCAGGCAGGTGGTGACACCCGACATGACGGCGG
 16 KhEt_2          80.3%  24.2%    GTTGACGGCCAACTTCCTCAGGTCAGAGTTCAACTGACCGGGGAAACGCAGGCAGGTGGTGACACCAGACATGACGGCGG
 17 KaEt_1          61.0%  24.9%    GTTGACGGCCAACTTCCTCAGGTCAGAGTTCAACTGACCGGGGAAACGCAGGCTGGTGGTGACACCCGACATGACGGCTG
 18 KhEt_5          81.4%  25.4%    GTTGACGGCCAACTTCCTCAGGTCAGAGTTCAACTGACCGGGGAAACGCAGGCAGGTGGTGACACCCGACATGACGGCGG
 19 KhEt_3          81.1%  24.6%    GTTGACGGCCAACTTCCTCAGGTCAGAGTTCAACTGACCGGGGAAACGCAGGCAGGTGGTGACACCCGACATGACGGCGG
 20 KhEt_4          82.5%  25.1%    GTTGACGGCCAACTTCCTCAGGTCAGAGTTCAACTGACCGGGGAAACGCAGGCAGGTGGTGACACCCGACATGACGGCGG
 21 KaEt_3          68.2%  24.9%    GTTGACGGCCAACTTCCTCAGGTCAGAGTTCAACTGACCGGGGAAACGCAGGCAGGTGGTGACACCCGACATGACGGCGG
 22 BhEt_9          57.5%  24.6%    GTTGACGGCCAACTTCCTCAGGTCAGAGTTCAACTGACCGGGGAAACGCAGGCAGGTGGTGACACCCGACATGACGGCGG
 23 BhEt_8          59.9%  25.3%    GTTGACGGCCAACTTCCTCAGGTCAGAGTTCAACTGACCGGGGAAACGCAGGCAGGTGGTGACACCCGACATGACGGCGG
 24 KaEt_6          82.7%  23.9%    GTTGACGGCGAGCTTTCGGAGGTCATAGTTCAGCTGACCGGGGAAACGGAGACAGGTGGTGACGCCGGACATGACGGCGG
 25 SaEt_5          91.0%  23.4%    GTTGACGGCGAGCTTTCGGAGGTCAGAGTTCAGCTGACCGGGGAAACGGAGACAGGTGGTGACGCCGGACATGACGGCGG
```

```
 26 KaEt_7          82.2%  24.1%      GTTGACGGCGAGCTTTCGGAGGTCAGAGTTCAGCTGACCGGGGAAACGGAGACAGGTGGTGACGCCGGACATGACGGCGG
 27 KaEt_4          82.4%  24.1%      GTTGACGGCGAGCTTTCGGAGGTCAGAGTTCAGCTGACCGGGGAAACGGAGACAGGTGGTGACGCCGGACATGACGGCGG
 28 KaEt_9          77.2%  24.2%      GTTGACGGCGAGCTTTCGGAGGTCAGAGTTCAGCTGACCGGGGAAACGGAGACAGGTGGTGACGCCGGACATGACGGCGG
 29 KaEt_8          81.9%  24.1%      GTTGACGGCGAGCTTTCGGAGGTCAGAGTTCAGCTGACCGGGGAAACGGAGACAGGTGGTGACGCCGGACATGACGGCGG
 30 BeEt_7          81.8%  23.9%      GTTGACGGCGAGCTTTCGGAGGTCAGAGTTCAGCTGACCGGGGAAACGGAGACAGGTGGTGACGCCGGACATGACGGCGG
 31 BeEt_3          82.1%  23.7%      GTTGACGGCGAGCTTTCGGAGGTCAGAGTTCAGCTGACCGGGGAAACGGAGACAGGTGGTGACGCCGGACATGACGGCGG
 32 KhEt_8          82.2%  23.8%      GTTGACGGCGAGCTTTCGGAGGTCAGAGTTCAGCTGACCGGGGAAACGGAGACAGGTGGTGACGCCGGACATGACGGCGG
 33 KhEt_7          78.5%  23.9%      GTTGACGGCGAGCTTTCGGAGGTCAGAGTTCAGCTGACCGGGGAAACGGAGACAGGTGGTGACGCCGGACATGACGGCGG
 34 BhEt_2          61.8%  22.7%      GTTGACGGCCAACTTCCTCAGGTCAGAGTTGAGCTGACCGGGGAAACGCATGCAGGTTGTGACACCCGACATGACAGCGG
 35 BhEt_1          52.4%  18.6%      GTTGACGGCCAACTTCCTCAGGTCAGAGTTGAGCTGACCGGGGAAACGCATGCAGGTTGTGACACCCGACATGACAGCGG
 36 BeEt_4          83.5%  26.0%      GTTGACGGCCAACTTCCTCAGGTCAGAGTTGAGCTGACCGGGGAAACGCAGGCG-GTAGTGACACCCGACATGACAGCGG
 37 SaEt_3          81.9%  25.0%      GTTGACGGCCAGCTTCCTCAGGTCAGAGTTGAGCTGACCAGGGAAACGCAGGCAGGTGGTGACACCCGACATGACGGCGG
 38 KhEt_9          82.7%  25.2%      GTTGACGGCCAGCTTCCTCAGGTCAGAGTTGAGCTGGCCTGGGAAACGCAGGCAGGTGGTGACACCCGACATGACGGCGG
 39 SaEt_4          82.7%  25.9%      GTTGACGGCCAACTTCCTCAGGTCAGAGTTGAGCTGACCGGGGAAACGCAGGCAGGTAGTGACACCCGACATGACAGCGG
 40 SaEt_8          91.0%  25.3%      GTTGACGGCCAGCTTCCTCAGGTCAGAGTTGAGCTGACCAGGGAAACGCAGGCAGGTGGTGACACCCGACATGACGGCGG
 41 KaEt_5          91.7%  25.0%      GTTGACGGCCAGCTTCCTCAGGTCAGAGTTGAGCTGACCAGGGAAACGCAGGCAGGTGGTGACACCCGACATGACGGCGG
 42 KaEt_2          82.2%  25.9%      GTTGACGGCCAGCTTCCTCAGGTCAGAGTTGAGCTGACCAGGGAAACGCAGACAGGTGGTGACACCCGACATGACGGCGG
 43 KhEt_6          82.5%  25.9%      GTTGACGGCCAGCTTCCTCAGGTCAGAGTTGAGCTGACCAGGGAAACGCAGACAGGTGGTGACACCCGACATGACGGCGG
 44 SaEt_2          70.8%  26.0%      GTTGACGGCCAGCTTCCTCAGGTCAGAGTTGAGCTGACCAGGGAAACGCAGACAGGTGGTGACACCCGACATGACGGCGG
 45 SaEt_1          82.4%  25.9%      GTTGACGGCCAGCTTCCTCAGGTCAGAGTTGAGCTGACCAGGGAAACGCAGACAGGTGGTGACACCCGACATGACGGCGG
    consensus/100%                    ................................................................................
    consensus/90%                     GTTGACGGCsAuCTTsCssAGGTCAGAGTTsAuCTGACCuGGGAAACGsAsuCuGGTuGTuACuCCsGACATGACuGCuG
    consensus/80%                     GTTGACGGCsAuCTTsCssAGGTCAGAGTTsAuCTGACCuGGGAAACGsAGuCAGGTGGTGACuCCsGACATGACGGCGG
    consensus/70%                     GTTGACGGCsAuCTTsCssAGGTCAGAGTTsAuCTGACCuGGGAAACGsAGuCAGGTGGTGACACCsGACATGACGGCGG

                       cov    pid  641        :         .         .         .         .         7         .         . 720
  1 BhEt_3         100.0% 100.0%      CCACCAGCTTGTCGAGAACTCTGA-CGAGACCTTCTGTATCGACAACGAGGCCCTCTACGACATCTGCATGAGGACCCTC
  2 BeEt_2          60.7%  19.0%      ATCGAGGTAGTTGAGGGTCACCGTATGATGGGTTGGACAGCTTC---AGGTGCGCA-TGCATATTTCTTAGAGG-TCCTC
  3 BeEt_1          60.7%  19.7%      ATCGAGGTAGTTGAGGGTCACCGTATGATGGGTTGGACAGCTTC---AGGTGCGCA-TGCATATTTCTTAGAGG-TCCTC
  4 KhEt_1          82.5%  23.5%      AAACAAGGTGGTCCAGGTCCCCGTAAGAAGGGTTTGACGACTTG---AGAGTGCGCATGCAATGTCTAAA---AGGCCTC
  5 BeEt_9          94.5%  24.6%      AGACCAGGTAGTTCAGGTCACCGTACGTAGGGTTAGACAGCTTG---AGGGTTCTCATGCAGATATCGTACAGA-GCCTC
  6 SaEt_7          95.0%  23.9%      AGACCAGGTAGTTCAGGTCACCGTACGTAGGGTTAGACAGCTTG---AGGGTTCTCATGCAGATATCGTACAGA-GCCTC
  7 SaEt_6          92.6%  24.3%      AGACCAGGTAGTTCAGGTCACCGTACGTAGGGTTAGACAGCTTG---AGGGTTCTCATGCAGATATCGTACAGA-GCCTC
  8 SaEt_9          93.9%  23.7%      AGACCAGGTAGTTCAGGTCACCGTACGTAGGGTTAGACAGCTTG---AGGGTTCTCATGCAGATATCGTACAGA-GCCTC
  9 BeEt_8          93.2%  22.9%      AGACCAGGTAGTTCAGGTCACCGTACGTAGGGTTAGACAGCTTG---AGGGTTCTCATGCAGATATCGTACAGA-GCCTC
 10 BeEt_6          82.5%  22.4%      AGACCAGGTAGTTCAGGTCACCGTACGTAGGGTTAGACAGCTTG---AGGGTTCTCATGCAGATATCGTACAGA-GCCTC
 11 BeEt_5          92.9%  24.2%      AGACCAGGTAGTTCAGGTCACCGTACGTAGGGTTAGACAGCTTG---AGGGTTCTCATGCAGATATCGTACAGA-GCCTC
 12 BhEt_7          82.5%  24.1%      -------------------------------GGGGTTGAGCTTG---AGAGTCCTCATGCAGATGTCGTAGAGG-GCCTC
 13 BhEt_6          84.6%  23.2%      AGACGAGGTGGTTCAGGTCACCGTAGGAGGGGTTGTTGAGCTTG---AGAGTCCTCATGCAGATGTCGTAGAGG-GCCTC
 14 BhEt_5          88.4%  24.2%      AGACGAGGTGGTTCAGGTCACCGTAGGAGGGGTTGTTGAGCTTG---AGAGTCCTCATGCAGATGTCGTAGAGG-GCCTC
 15 BhEt_4          90.9%  24.9%      AGACGAGGTGGTTCAGGTCACCGTAGGAGGGGTTGTTGAGCTTG---AGAGTCCTCATGCAGATGTCGTAGAGG-GCCTC
 16 KhEt_2          80.3%  24.2%      AGACGAGGTGGTTCAGGTCACCGTAGGAGGGGTTGTTGAGCTTG---AGAGTCCTCATGCAGATGTCGTATAGG-GCCTC
 17 KaEt_1          61.0%  24.9%      A-ACGAGGTGGTTCAGGTCACCGTAGGAGGGGTTGTTGAGCTTG---AGAGTCCTCATGCAGATTTCGTAGAGGGGCCTC
 18 KhEt_5          81.4%  25.4%      AGACGAGGTGGTTCAGGTCACCGTAGGAGGGGTTGTTGAGCTTG---AGGGTCCTCATGCAGATGTCGTAGAGG-GCCTC
 19 KhEt_3          81.1%  24.6%      AGACGAGGTGGTTCAGGTCACCGTAGGAGGGGTTGTTGAGCTTG---AGGGTCCTCATGCAGATGTCGTAGAGG-GCCTC
 20 KhEt_4          82.5%  25.1%      AGACGAGGTGGTTCAGGTCACCGTAGGAGGGGTTGTTGAGCTTG---AGAGTCCTCATGCAGATGTCGTAGAGG-GCCTC
 21 KaEt_3          68.2%  24.9%      AGACGAGGTGGTTCAGGTCACCGTAGGAGGGGTTGTTGAGCTTG---AGGGTCCTCATGCAGATGTCGTAGAGG-GCCTC
 22 BhEt_9          57.5%  24.6%      AGACGAGGTGGTTCAGGTCACCGTAGGAGGGGTTGTTGAGCTTG---AGGGTCCTCATGCAGATGTCGTAGAGG-GCCTC
 23 BhEt_8          59.9%  25.3%      AGACGAGGTGGTTCAGGTCACCGTAGGAGGGGTTGTTGAGCTTG---AGGGTCCTCATGCAGATGTCGTAGAGG-GCCTC
 24 KaEt_6          82.7%  23.9%      AGACTAGGTATTTCAAGTCGCCGTAAGAGGGGTTGGACAGCTTG---AGGGTGCGCATGCAGATGTCGTAAAGG-GCCTC
 25 SaEt_5          91.0%  23.4%      AGACGAGGTAGTTCAAGTCGCCGTAAGAGGGGTTGGACAGCTTG---AGGGTGCGCATGCAGATGTCGTAAAGG-GCCTC
 26 KaEt_7          82.2%  24.1%      AGACGAGGTAGTTCAAGTCGCCGTAAGAGGGGTTGGACAGCTTG---AGGGTGCGCATGCAGATGTCGTAAAGG-GCCTC
 27 KaEt_4          82.4%  24.1%      AGACGAGGTAGTTCAAGTCGCCGTAAGAGGGGTTGGACAGCTTG---AGGGTGCGCATGCAGATGTCGTAAAGG-GCCTC
 28 KaEt_9          77.2%  24.2%      AGACGAGGTAGTTCAAGTCGCCGTAAGAGGGGTTGGACAGCTTG---AGGGTGCGCATGCAGATGTCGTAAAGG-GCCTC
 29 KaEt_8          81.9%  24.1%      AGACGAGGTAGTTCAAGTCGCCGTAAGAGGGGTTGGACAGCTTG---AGGGTGCGCATGCAGATGTCGTAAAGG-GCCTC
 30 BeEt_7          81.8%  23.9%      AGACGAGGTAGTTCAAGTCGCCGTAAGAGGGGTTGGACAGCTTG---AGGGTGCGCATGCAGATGTCGTAAAGG-GCCTC
 31 BeEt_3          82.1%  23.7%      AGACGAGGTAGTTCAAGTCGCCGTAAGAGGGGTTGGACAGCTTG---AGGGTGCGCATGCAGATGTCGTAAAGG-GCCTC
 32 KhEt_8          82.2%  23.8%      AGACGAGGTAGTTCAAGTCGCCGTAAGAGGGGTTGGACAGCTTG---AGGGTGCGCATGCAGATGTCGTAAAGG-GCCTC
 33 KhEt_7          78.5%  23.9%      AGACGAGGTAGTTCAAGTCGCCGTAAGAGGGGTTGGACAGCTTG---AGGGTGCGCATGCAGATGTCGTAAAGG-GCCTC
 34 BhEt_2          61.8%  22.7%      AAACGAGGTGGTTCAGGTCGCCGTAGGAGGGGTTGTTCATCTTG---AGAGTCCTCATGCCGATTCG-TATAAA-GCCTT
 35 BhEt_1          52.4%  18.6%      AAACGAGGTGGTTCAGGTCGCCGTAGGAGGGGTTGTTCATCTTG---AGAGTCCTCATGCCGATTCG-TATAAA-GCCTT
 36 BeEt_4          83.5%  26.0%      AAACGAGGTGGTTCAGGTCGCCGTA-GAGGGGTTGTTCAGCTTG---AGAGTCCTCATGCAGATGTCGTAGAGA-GCCTC
 37 SaEt_3          81.9%  25.0%      ATACGAGGTGGTTCAGGTCACCGTACTAGGGGTTGTTCAGCTTG---AGGGTCCTCATGCAAATGTCGTAGAGA-GCCTC
 38 KhEt_9          82.7%  25.2%      AAACGAGGTGGTTCAGGTCACCGTACGAGGGGTTGTTCAGCTTG---AGGGTCCTCATGCAAATGTCGTAGAGA-GCCTC
 39 SaEt_4          82.7%  25.9%      A-ACGAGGTGGTTCAGGTCGCCGTAGGAGGGGTTGTTCAGCTTG---AGAGTCCTCATGCAGATGTCGTAGAGA-GCCTC
 40 SaEt_8          91.0%  25.3%      AGACGAGGTGGTTCAGGTCACCGTACGAGGGGTTGTTCAGCTTG---AGGGTCCTCATGCAAATGTCGTAGAGA-GCCTC
 41 KaEt_5          91.7%  25.0%      AGACGAGGTGGTTCAGGTCACCGTACGAGGGGTTGTTCAGCTTG---AGGGTCCTCATGCAAATGTCGTAGAGA-GCCTC
 42 KaEt_2          82.2%  25.9%      AGACGAGGTGGTTCAGGTCGCCGTAGGAGGGGTTGTTCAGCTTG---AGGGTCCTCATGCAGATGTCGTAGAGA-GCCTC
 43 KhEt_6          82.5%  25.9%      AGACGAGGTGGTTCAGGTCGCCGTAGGAGGGGTTGTTCAGCTTG---AGGGTCCTCATGCAGATGTCGTAGAGA-GCCTC
 44 SaEt_2          70.8%  26.0%      AGACGAGGTGGTTCAGGTCGCCGTAGGAGGGGTTGTTCAGCTTG---AGGGTCCTCATGCAGATGTCGTAGAGA-GCCTC
 45 SaEt_1          82.4%  25.9%      AGACGAGGTGGTTCAGGTCGCCGTAGGAGGGGTTGTTCAGCTTG---AGGGTCCTCATGCAGATGTCGTAGAGA-GCCTC
    consensus/100%                    ...............................sssssssusCsss...uuussssss.ssssssssss.ss...u.sCCTs
    consensus/90%                     AsACsAGGTuGTsCAuGTCuCCGTAsGsuGGGTTusssAGCTTG...AGuGTsCsCATGCAuATsTCsTAsAGu.GCCTC
    consensus/80%                     AuACsAGGTuGTTCAuGTCuCCGTAsGAuGGGTTGsssAGCTTG...AGuGTsCsCATGCAGATuTCGTAsAGu.GCCTC
    consensus/70%                     AGACGAGGTuGTTCAGGTCuCCGTAsGAGGGGTTGssCAGCTTG...AGGGTsCTCATGCAGATuTCGTAuAGu.GCCTC

                       cov    pid  721        .         .         :         .         .         .         .         8 800
  1 BhEt_3         100.0% 100.0%      AA----GCTCAACAACCCCTCCTACGGTGACCTGAACCACCTCGTCTCCGCCGTCATGTCGGGTGTCAC------CACCT
```

```
  2 BeEt_2          60.7%  19.0%      TTTATCGATTC-TAATGTCTCTTCGGAGTTCTCTACCATCTTT---GAACGGATATGTTGTTTTTTTTGTCTCATCTACT
  3 BeEt_1          60.7%  19.7%      TTTATCGATTC-TAATGTCTCTTCGGAGTTCTCTACCATCTTT---GAACGGATATGTTGTTTTTTTTGTCTCATCTACT
  4 KhEt_1          82.5%  23.5%      GTTATCCATACAAAAGGTCTCGTCCGATT--CTCGACCAACTTGTGGACTGAAAGAGTAGCGTTGTAAGGCTCAACAACG
  5 BeEt_9          94.5%  24.6%      GTTGTCGATACAGAAGGTCTCGTCGGAGTTC-TCGACAAGCTGGTGAACGGAGAGAGTGGCGTTGTAAGGCTCAACAACG
  6 SaEt_7          95.0%  23.9%      GTTGTCGATACAGAAGGTCTCGTCGGAGTTC-TCGACAAGCTGGTGAACGGAGAGAGTGGCGTTGTAAGGCTCAACAACG
  7 SaEt_6          92.6%  24.3%      GTTGTCGATACAGAAGGTCTCGTCGGAGTTC-TCGACAAGCTGGTGAACGGAGAGAGTGGCGTTGTAAGGCTCAACAACG
  8 SaEt_9          93.9%  23.7%      GTTGTCGATACAGAAGGTCTCGTCGGAGTTC-TCGACAAGCTGGTGAACGGAGAGAGTGGCGTTGTAAGGCTCAACAACG
  9 BeEt_8          93.2%  22.9%      GTTGTCGATACAGAAGGTCTCGTCGGAGTTC-TCGACAAGCTGGTGAACGGAGAGAGTGGCGTTGTAAGGCTCAACAACG
 10 BeEt_6          82.5%  22.4%      GTTGTCGATACAGAAGGTCTCGTCGGAGTTC-TCGACAAGCTGGTGAACGGAGAGAGTGGCGTTGTAAGGCTCAACAACG
 11 BeEt_5          92.9%  24.2%      GTTGTCGATACAGAAGGTCTCGTCGGAGTTC-TCGACAAGCTGGTGAACGGAGAGAGTGGCGTTGTAAGGCTCAACAACG
 12 BhEt_7          82.5%  24.1%      GTTGTCGATACAGAAGGTCTCGTCAGAGTTC-TCGACAAGCTGGTGGATGGAGAGGGTGGCGTTGTAGGGCTCGACAACG
 13 BhEt_6          84.6%  23.2%      GTTGTCGATACAGAAGGTCTCGTCAGAGTTC-TCGACAAGCTGGTGGATGGAGAGGGTGGCGTTGTAGGGCTCGACAACG
 14 BhEt_5          88.4%  24.2%      GTTGTCGATACAGAAGGTCTCGTCAGAGTTC-TCGACAAGCTGGTGGATGGAGAGGGTGGCGTTGTAGGGCTCGACAACG
 15 BhEt_4          90.9%  24.9%      GTTGTCGATACAGAAGGTCTCGTCAGAGTTC-TCGACAAGCTGGTGGATGGAGAGGGTGGCGTTGTAGGGCTCGACAACG
 16 KhEt_2          80.3%  24.2%      GTTGTCGATACAGAAGGTCTCGTCAGAGTTC-TCAACGAGCTGGTGGATGGAGAGGGTGGCGTTGTATGGCTCTTCGACG
 17 KaEt_1          61.0%  24.9%      GTTGTCGATACAGAATGTCTCGTCAGAGTTC-TCGACTAGCTGGTGGATGGAGAGGGTGGCGTTGTATGGCTCGATAACG
 18 KhEt_5          81.4%  25.4%      GTTGTCGATACAGAAGGTCTCGTCAGAGTTC-TCGACAAGCTGGTGGATGGAGAGAGTGGCGTTGTAGGGCTCGACAACG
 19 KhEt_3          81.1%  24.6%      GTTGTCGATACAGAAGGTCTCGTCAGAGTTC-TCGACAAGCTGGTGGATGGAGAGGGTGGCGTTGTAGGGCTCGACAACG
 20 KhEt_4          82.5%  25.1%      GTTGTCGATACAGAAGGTCTCGTCAGAGTTC-TCGACAAGCTGGTGGATGGAGAGGGTGGCGTTGTAGGGCTCGACAACG
 21 KaEt_3          68.2%  24.9%      GTTGTCGATACAGAAGGTCTCGTCAGAGTTC-TCGACAAGCTGGTGGATGGAGAGGGTGGCGTTGTAGGGCTCGACAACT
 22 BhEt_9          57.5%  24.6%      GTTGTCGATACAGAAGGTCTCGTCAGAGTTC-TCGACAAGCTGGTGGATGGAGAGGGTTGCGTTGTATG-----------
 23 BhEt_8          59.9%  25.3%      GTTGTCGATACAGAAGGTCTCGTCAGAGTTC-TCGACAAGCTGGTGGATGGAGAGGGTTGCGTTGTATG-----------
 24 KaEt_6          82.7%  23.9%      GTTATCGATACAGAAAGTCTCGTCGGAGTTC-TCGACCAGCTGGTGGACGGAAAGAGTAGCGTTGTAGGGCTCAACAACG
 25 SaEt_5          91.0%  23.4%      GTTATCGATACAGAAAGTCTCGTCGGAGTTC-TCGACCAGCTGGTGGACGGAAAGAGTAGCGTTGTAGGGCTCAACAACG
 26 KaEt_7          82.2%  24.1%      GTTATCGATACAGAAAGTCTCGTCGGAGTTC-TCGACCAGCTGGTGGACGGAAAGAGTAGCGTTGTAGGGCTCAACAACG
 27 KaEt_4          82.4%  24.1%      GTTATCGATACAGAAAGTCTCGTCGGAGTTC-TCGACCAGCTGGTGGACGGAAAGAGTAGCGTTGTAGGGCTCAACAACG
 28 KaEt_9          77.2%  24.2%      GTTATCGATACAGAAAGTCTCGTCGGAGTTC-TCGACCAGCTGGTGGACGGAAAGAGTAGCGTTGTAGGGCTCAACAACG
 29 KaEt_8          81.9%  24.1%      GTTATCGATACAGAAAGTCTCGTCGGAGTTC-TCGACCAGCTGGTGGACGGAAAGAGTAGCGTTGTAGGGCTCAACAACG
 30 BeEt_7          81.8%  23.9%      GTTATCGATACAGAAAGTCTCGTCGGAGTTC-TCGACCAGCTGGTGGACGGAAAGAGTAGCGTTGTAGGGCTCAACAACG
 31 BeEt_3          82.1%  23.7%      GTTATCGATACAGAAAGTCTCGTCGGAGTTC-TCGACCAGCTGGTGGACGGAAAGAGTAGCGTTGTAGGGCTCAACAACG
 32 KhEt_8          82.2%  23.8%      GTTATCGATACAGAAAGTCTCGTCGGAGTTC-TCGACCAGCTGGTGGACGGAAAGAGTAGCGTTGTAGGGCTCAACAACG
 33 KhEt_7          78.5%  23.9%      GTTATCGATACAGAAAGTCTCGTCGGAGTTC-TCGACCAGCTGGTGGACGGAAAGAGTAGCGTTGTAGGGCTCAACAACG
 34 BhEt_2          61.8%  22.7%      GTTGTCTATGCAGAAGGTCTCGTCCGACTTCTCCCCCATCTCTTTATGGATATTGTTCTGTTTTTTTTTTTTTTGCTGCTC
 35 BhEt_1          52.4%  18.6%      GTTGTCTATGCAGAAGGTCTCGTCCGACTTCTCCCCCATCTCTTTATGGATATTGTTCTGTTTTTTTTTTTTTTGCTGCTC
 36 BeEt_4          83.5%  26.0%      GTTGTCAATGCAGAAGGTCTCGTCCGAGT-TCTCAACCAGCTGGTGGATGGAGAGTGTGGCGTTGTAGGGCTCGACAACA
 37 SaEt_3          81.9%  25.0%      GTTGTCAATGCAGAAGGTCTCGTCCGAGTTTCTCAACCAGCTGGTGGATGGAGAGTGTGGTGTTGTAGGGCTCGATTACG
 38 KhEt_9          82.7%  25.2%      GTTGTCAATGCAGAAGGTCTCGTCAGAGT-TCTCAACCAGCTGGTGGATGGAGAGTGTGGCGTTGTAGGGCTCGACAACG
 39 SaEt_4          82.7%  25.9%      GTTGTCAATGCAGAAGGTCTCGTCCGAGT-TCTCAACCAGCTGGTGGATGGAGAGTGTGGCGTTGTAGGGCTCGACAACG
 40 SaEt_8          91.0%  25.3%      GTTGTCAATGCAGAAGGTCTCGTCCGAGT-TCTCAACCAGCTGGTGGATGGAGAGTGTGGCGTTGTAGGGCTCGACAACG
 41 KaEt_5          91.7%  25.0%      GTTGTCAATGCAGAAGGTCTCGTCCGAGT-TCTCAACCAGCTGGTGGATGGAGAGTGTGGCGTTGTAGGGCTCGACAACG
 42 KaEt_2          82.2%  25.9%      GTTGTCAATGCAGAAGGTCTCGTCGGAGT-TCTCAACCAGCTGGTGGATGGAGAGTGTGGCGTTGTAGGGCTCGACA-CG
 43 KhEt_6          82.5%  25.9%      GTTGTCAATGCAGAAGGTCTCGTCGGAGT-TCTCAACCAGCTGGTGGATGGAGAGTGTGGCGTTGTAGGGCTCGACAACG
 44 SaEt_2          70.8%  26.0%      GTTGTCAATGCAGAAGGTCTCGTCGGAGT-TCTCAACCAGCTGGTGGATGGAGAGTGTGGCGTTGTAGGGCTCGACAACG
 45 SaEt_1          82.4%  25.9%      GTTGTCAATGCAGAAGGTCTCGTCGGAGT-TCTCAACCAGCTGGTGGATGGAGAGTGTGGCGTTGTAGGGCTCGACAACG
    consensus/100%                    ss....ssTss.sAAsssCTCsTssGuss...ssssCssssss...ssssssssssssssssTsTsss...........
    consensus/90%                     GTTuTCuATuCAGAAuGTCTCGTCsGAGT.s.TCuACsAsCTsGTuuAsuGAusGsGTsGsGTTGTssGssTsussssCs
    consensus/80%                     GTTuTCuATuCAGAAuGTCTCGTCuGAGT.s.TCuACsAGCTGGTGuAsGGAuAGsGTuGCGTTGTAuGGCTCuACAACG
    consensus/70%                     GTTuTCGATuCAGAAuGTCTCGTCuGAGTTC.TCuACsAGCTGGTGGAsGGAuAGsGTuGCGTTGTAuGGCTCuACAACG

                     cov    pid  801         .         .         .         .         :         .         .         . 880
  1 BhEt_3         100.0% 100.0%      GCCT--------------------------------------GCGTTTCCCCGGTCAGTTGAACTCTGACCTGAGGAAGT
  2 BeEt_2          60.7%  19.0%      GTGGCATATACCTGTGTGGGATGTATCTACTAGATTT-------------------------------------------
  3 BeEt_1          60.7%  19.7%      GTGGCATATACCTGTGTGGGATGTATCTACTAGATTT-------------------------------------------
  4 KhEt_1          82.5%  23.5%      GTGTCGGAGACCTTAGGAGAAGGGACGACGAAAGTAGCCA------TCATACG--TCGGGGAACTCCTCACGGATCTTGG
  5 BeEt_9          94.5%  24.6%      GTGTCGGAAACCTTGGGGGGGAGGGGGACAACGGAGAAGGTAGCCATCATGCGGTCGGGGAACTCCTTCACGAAATCTT
  6 SaEt_7          95.0%  23.9%      GTGTCGGAAACCTTGGGGGGAG--GGGACAACGGAGAAGGTAGCCATCATGCGGTCGGGGA-ACTCCTCACGAAATCTTG
  7 SaEt_6          92.6%  24.3%      GTGTCGGAAACCTTGGGGGGAG--GGGACAACGGAGAAGGTAGCCATCATGCGGTCGGGGA-ACTCCTCACGAAATCTTG
  8 SaEt_9          93.9%  23.7%      GTGTCGGAAACCTTGGGGGGAG--GGGACAACGGAGAAGGTAGCCATCATGCGGTCGGGGGAACTCCTCACGAAATCTTG
  9 BeEt_8          93.2%  22.9%      GTGTCGGAAACCTTGGGGGGAG--GGGACAACGGAGAAGGTAGCCATCATGCGGTCGGGGGAACTCCTCACGAATCTT--
 10 BeEt_6          82.5%  22.4%      GTGTCGGAAACCTTGGGGGGAG--GGGACAACGGAGAAGGTAGCCATCATGCGGTCGGGGGAACTCCTCACGAATCTT--
 11 BeEt_5          92.9%  24.2%      GTGTCGGAAACCTTGGGGGGAG--GGGACAACGGAGAAGGTAGCCATCATGCGGTCGGGGGAACTCCTCACGAATCTT--
 12 BhEt_7          82.5%  24.1%      GTGTCGGAGACCTTGGGGGAGGGCACAACGGAGAAGGTGG--CCAT-CATGCGG-TCGGGGAACTCCTCGCGGATCTTGG
 13 BhEt_6          84.6%  23.2%      GTGTCGGAGACCTTGGGGGAGGGCACAACGGAGAAGGTGG--CCAT-CATGCGG-TCGGGGAA-----------------
 14 BhEt_5          88.4%  24.2%      GTGTCGGAGACCTTGGGGGAGGGCACAACGGAGAAGGTGG--CCAT-CATGCGG-TCGGGGAACTCCTCGCGGATCTTGG
 15 BhEt_4          90.9%  24.9%      GTGTCGGAGACCTTGGGGGAGGGCACAACGGAGAAGGTGG--CCAT-CATGCGG-TCGGGGAACTCCTCGCGGATCTTGG
 16 KhEt_2          80.3%  24.2%      GTTTCGGAGATCTTTGGTGGAGGGGTTTACGATATGCGCG--TCTCGTCTGTCCGTCTGGGGACGCCTCACGGATGTTGT
 17 KaEt_1          61.0%  24.9%      GG------------------------------------------------------------------------------
 18 KhEt_5          81.4%  25.4%      GTGTCGGAGACCTTGGGGGAGGGCACAACGGAGAAGGTGG--CC-ATCATGCGGTCGGGGAA-CTCCTCGCGGATCTTGG
 19 KhEt_3          81.1%  24.6%      GTGTCGGATACCTTGGGGGAGTCACTACTGGATAATGTGG--CC-ATCATGCGGTCGTGGGATGTCCTCTCGGATCTTGG
 20 KhEt_4          82.5%  25.1%      GTGTCGGAACC-TTGGGGGAGGGCACAACGGAGAAGGTGG--CC-ATCATGCGGTCGGGAA-ACTCCTCGCGGATCTTGG
 21 KaEt_3          68.2%  24.9%      GTGTCGGAGACCTTGGGGGAGTGCACAACGGAGAAGGTGG--CC-ATCACGCGGTCGGGGA-ACTCCTCGCGTATCTTGG
 22 BhEt_9          57.5%  24.6%      --------------------------------------------------------------------------------
 23 BhEt_8          59.9%  25.3%      --------------------------------------------------------------------------------
 24 KaEt_6          82.7%  23.9%      GTGTCGGAGACCTTAGGAGAA---GGGACGACGGAGAAGGTATCCATCGTACGGTCGGG-GAACTCCTCACGGATCTTG-
 25 SaEt_5          91.0%  23.4%      GTGTCGGAGACCTTAGGAGAA---GGGACGACGGAGAAGGTAGCCATCATACGGTCGGG-GAACTCCTCACGGATCTTGG
 26 KaEt_7          82.2%  24.1%      GTGTCGGAGACCTTAGGAGAA---GGGACGACGGAGAAGGTAGCCATCATACGGTCGGG-GAACTCCTCACGGATCTTGG
 27 KaEt_4          82.4%  24.1%      GTGTCGGAGACCTTAGGAGAA---GGGACGACGGAGAAGGTAGCCATCATACGGTCGGG-GAACTCCTCACGGATCTTGG
 28 KaEt_9          77.2%  24.2%      GTGTCGGAGACCTTAGGAGAA---GGGACGACGGAGAAGGTAGCCATCATACGGTCGGG-GAACTCCTCACGGATCTTGG
```

```
29 KaEt_8          81.9%  24.1%      GTGTCGGAGACCTTAGGAGAA---GGGACGACGGAGAAGGTAGCCATCATACGGTCGGG-GAACTCCTCACGGATCTTGG
30 BeEt_7          81.8%  23.9%      GTGTCGGAGACCTTAGGAGAA---GGGACGACGGAGAAGGTAGCCATCATACGGTCGGG-GAACTCCTCACGGATCTTGG
31 BeEt_3          82.1%  23.7%      GTGTCGGAGACCTTAGGAGAA---GGGACGACGGAGAAGGTAGCCATCATACGGTCGGG-GAACTCCTCACGGATCTTGG
32 KhEt_8          82.2%  23.8%      GTGTCGGAGACCTTAGGAGAA---GGGACGACGGAGAAGGTAGCCATCATACGGTCGGG-GAACTCCTCACGGATCTTGG
33 KhEt_7          78.5%  23.9%      GTGTCGGAGACCTTAGGAGAA---GGGACGACGGAGAAGGTAGCCATCATACGGTCGGG-GAACTCCTCACGGATCTTGG
34 BhEt_2          61.8%  22.7%      CCCTCTTTTTTATTCATGCTTCACTTCTCCTCCCTTTCTCTGCTC-----------------------------------
35 BhEt_1          52.4%  18.6%      CCCTCTTTTTTATTCATGCTTCACTTCTCCTCCCTTTCTCTGCTC-----------------------------------
36 BeEt_4          83.5%  26.0%      GTGTCAGATATCTTGGGGGAA---GGCACGACGGCCTACGTGGCCGTCATGCGATCTGGTA-ACTCCTCTCGGATCTGGG
37 SaEt_3          81.9%  25.0%      GTGTCGTAGACCTTGGGCGAG---GGCACGACCGAGTAGGTGGCCATCATACGGTCGGGGGAACTCCTCACGGGATCTTG
38 KhEt_9          82.7%  25.2%      GTGTCGGAGACCTTGGGAGAG---GGCACGACCGAGTATGTGGCCATCATACGGTCGGGGTGACTCCTCACTGATCTTGG
39 SaEt_4          82.7%  25.9%      GTGTCGGAGACCTTGGGGGAA---GGCACGACGGAGTAGGTGGCCATCATGCGGTCGGGGAACTCC--TCACGGATCTGG
40 SaEt_8          91.0%  25.3%      GTGTCGGAGACCTTGGGGCGA--GGGCACGACCGAGTAGGTGGCCATCATGCGGTCGGGGGAACTCCTCACGGATCTTGG
41 KaEt_5          91.7%  25.0%      GTGTCGGAGACCTTGGG-CGA--GGGCACGACCGAGTAGGTGGCCATCATGCGGTCG-GGGAACTCCTCACGGATCTTGG
42 KaEt_2          82.2%  25.9%      GTGTCGGAGACCTTGGG-CGA--GGGCACAACGGAGTATGTGGCCATCATGCGGTCG-GGGAACTCCTCACGGATCTTGG
43 KhEt_6          82.5%  25.9%      GTGTCGGAGACCTTGGG-CGA--GGGCACAACGGAGTATGTGGCCATCATGCGGTCG-GGGAACTCCTCACGGATCTTGG
44 SaEt_2          70.8%  26.0%      GTGTCGGAGACCTTGGG-CGA--GGGCACAACGGAGTATGTGGCCATCATGCGGTCG-GGGAACTCCTCACGGATCTTGG
45 SaEt_1          82.4%  25.9%      GTGTCGGAGACCTTGGG-CGA--GGGCACAACGGAGTATGTGGCCATCATGCGGTCG-GGGAACTCCTCACGGATCTTGG
   consensus/100%                    ................................................................................
   consensus/90%                     GsssCssssss.Tssus.sss...sssssssssssss...........................................
   consensus/80%                     GTGTCGGAsACCTTuGGssuu...usssCuussusssssG..sC.s.CuTuCGu.ss.G.u.sssCssssssuussss..
   consensus/70%                     GTGTCGGAuACCTTuGGuGuu...ussACuusGuAGsssG..sCssTCATuCGGTCssG.uuACTCCTCuCGuAsssTss

                    cov    pid 881         .         9         .         .         .         .         :         . 960
 1 BhEt_3         100.0% 100.0%      TGGCCGTCAACATGGTTCCCTTCCCCCG-----------TCTCCACTTCTTCATGGTCGGTTTCGCTCCTCTGACCAGCC
 2 BeEt_2          60.7%  19.0%      --------------------------------------------------------------------------------
 3 BeEt_1          60.7%  19.7%      --------------------------------------------------------------------------------
 4 KhEt_1          82.5%  23.5%      AGA-TC--AGCAACGTACCCATACCGGCACCG-------GTACCACCCACCGAGAGAG-TGGGTGATCTGGAA---ACCC
 5 BeEt_9          94.5%  24.6%      GGA-GATAAGCAGAAGTTACCCATTACCCGGCCACCCGGTACCCACCACCCGAGGAGAGTGGGTGATTCTTGGAAACCCC
 6 SaEt_7          95.0%  23.9%      GGA-GATAAGCAGAGTAACCCATACCGGC-----ACCGGGTACCACCCACCGAAGAGAGTGGGTGATCT-GGGAAACCCC
 7 SaEt_6          92.6%  24.3%      GGA-GATAAGCAGAGTAACCCATACCGGC-----ACCGGGTACCACCCACCGAAGAGAGTGGGTGATCT-GGGAAACCCC
 8 SaEt_9          93.9%  23.7%      GGA-GATAAGCAGAGTACCCATACCGGCA-----CCGG-TACCA--CCACCGAGAGAGTGGGGTGATCT-GGAAAACCCC
 9 BeEt_8          93.2%  22.9%      GGA-GATAAGCAGAGTACCCATACCGGCA-----CCGG-TACCA--CCACCGAGAGAGTGGGTGATCTG-GAAACCCTGG
10 BeEt_6          82.5%  22.4%      GGA-GATAAGCAGAGTACCCATACCGGCA-----CCGG-TACCA--CCACCGAGAGAGTGGGTGATCTG-GAAAACCCCT
11 BeEt_5          92.9%  24.2%      GGA-GATAAGCAGAGTACCCATACCGGCA-----CCGG-TACCA--CCACCGAGAGAGTGGGTGATCTG-GAAAACCCCT
12 BhEt_7          82.5%  24.1%      AGA-TCAGGAGAG--TACCCATACCGGCACCG-------GTACCACC-ACCGAGGGAG-TGGGTGATCTGGAA-ACC--C
13 BhEt_6          84.6%  23.2%      --------------------------GCACCG-------GTACCACC-ACCGAGGGAG-TGGGTGATCTGGAA-ACC--C
14 BhEt_5          88.4%  24.2%      AGA-TCAGGAGAG--TACCCATACCGGCACCG-------GTACCACC-ACCGAGGGAG-TGGGTGATCTGGAA-ACC--C
15 BhEt_4          90.9%  24.9%      AGA-TCAGGAGAG--TACCCATACCGGCACCG-------GTACCACC-ACCGAGGGAG-TGGGTGATCTGGAA-ACC--C
16 KhEt_2          80.3%  24.2%      AGA-TGAAAGATA-GTACCGTTATGTTGCGTC-------GGTTTCGC-CCACTTGTTTTTGGGTGTTTTTGAA-TCCCGT
17 KaEt_1          61.0%  24.9%      --------------------------------------------------------------------------------
18 KhEt_5          81.4%  25.4%      AGA-TAA-GAGAG--TACCCATACCGGCACCG-------GTTCCACC-ACCGAGGGAGT-GGGTGATCTGGAA-AC--CC
19 KhEt_3          81.1%  24.6%      AGA-TGATGATAG--TACTCATTCCTGTCTCG-------GGTTCACC-TCCGAGGTGATTGTGTTATCTGGGA-AATCTT
20 KhEt_4          82.5%  25.1%      AGA-TAATGAGAG--TACCCATACCGGCACCG-------GTTCCACC-ACCGAGGGAG-TGGGTGATCTGGAA-ACCCTG
21 KaEt_3          68.2%  24.9%      AGA-TGAGG-----------------------------------------------------------------------
22 BhEt_9          57.5%  24.6%      --------------------------------------------------------------------------------
23 BhEt_8          59.9%  25.3%      --------------------------------------------------------------------------------
24 KaEt_6          82.7%  23.9%      AGA-TC--ATCAGCGTACCCATACCGGCACCG-------GTACCACC-ACCGAGAGAGTGT-GTGATCTG--G-AAACCC
25 SaEt_5          91.0%  23.4%      AGA-TC--AGCAGCGTACCCATACCGGCACCG-------GTACCACC-ACCGAGAGAGTGG-GTGATCTG--G-AAACCC
26 KaEt_7          82.2%  24.1%      AGA-TC--AGCAGCGTACCCATACCGGCACCG-------GTACCACC-ACCGAGAGAGTGG-GTGATCTG--G-AAACCC
27 KaEt_4          82.4%  24.1%      AGA-TC--AGCAGCGTACCCATACCGGCACCG-------GTACCACC-ACCGAGAGAGTGG-GTGATCTG--G-AAACCC
28 KaEt_9          77.2%  24.2%      AGA-TC--AGCAGCGTACCCATACCGGCACCG-------GTACCACC-ACCGAGAGAGTGG-GTGATCTG--G-AAACCC
29 KaEt_8          81.9%  24.1%      AGA-TC--AGCAGCGTACCCATACCGGCACCG-------GTACCACC-ACCGAGAGAGTGG-GTGATCTG--G-AAACCC
30 BeEt_7          81.8%  23.9%      AGA-TC--AGCAGCGTACCCATACCGGCACCG-------GTACCACC-ACCGAGAGAGTGG-GTGATCTG--G-AAACCC
31 BeEt_3          82.1%  23.7%      AGA-TC--AGCAGCGTACCCATACCGGCACCG-------GTACCACC-ACCGAGAGAGTGG-GTGATCTG--G-AAACCC
32 KhEt_8          82.2%  23.8%      AGA-TC--AGCAGCGTACCCATACCGGCACCG-------GTACCACC-ACCGAGAGAGTGG-GTGATCTG--G-AAACCC
33 KhEt_7          78.5%  23.9%      AGA-TC--AGCAGCGTACCCATACCGGCACCG-------GTACCACC-ACCGAGAGAGTGG-GTGATCTG--G-AAACCC
34 BhEt_2          61.8%  22.7%      --------------------------------------------------------------------------------
35 BhEt_1          52.4%  18.6%      --------------------------------------------------------------------------------
36 BeEt_4          83.5%  26.0%      AGA-TG--ATAACCGTACCCTTACCGTCACCG-------GTGGCACC-TCCTAAGGAGTGG-GGTGATCT-GT-AAGCCC
37 SaEt_3          81.9%  25.0%      GAT-ATGAGGAGCGTTCCCCTTACCGGCACCA-------TTACCGCC-ACCCGAGAGAGTG-GGTGATCTGGA-AACCCC
38 KhEt_9          82.7%  25.2%      AAT-GA--GAAGCGTTCCCATACCGGGCACCG-------GTACCACC-ACCGAGAGAGTGG-GGTGATCTGGA-AAACCC
39 SaEt_4          82.7%  25.9%      AGA-TG--AGAAGCGTACCCATACCGGCACCA-------GTACCACC-TCCCAGGGAGTGG-GTGATC--TGG-AAACCC
40 SaEt_8          91.0%  25.3%      AGA-TG--AGGAGCGTTCCCATACCGGCACCA-------GTACCGCC-ACCGAGAGAGTGG-GTGATC--TGG-AAACCC
41 KaEt_5          91.7%  25.0%      AGA-TG--AGGAGCGTTCCCATACCGGCACCA-------GTACCGCC-ACCGAGAGAGTGG-GTGATC--TGG-AAACCC
42 KaEt_2          82.2%  25.9%      AAA-TG--AGGAGCGTTCCCATACCGGCACCG-------GTACCACC-ACCGAGAGAGTGG-GTGATC--TGG-AAACCC
43 KhEt_6          82.5%  25.9%      AAA-TG--AGGAGCGTTCCCATACCGGCACCG-------GTACCACC-ACCGAGAGAGTGG-GTGATC--TGG-AAACCC
44 SaEt_2          70.8%  26.0%      AAA-TG--AGGAGCGTTCCCATACCG------------------------------------------------------
45 SaEt_1          82.4%  25.9%      AAA-TG--AGGAGCGTTCCCATACCGGCACCG-------GTACCACC-ACCGAGAGAGTGG-GTGATC--TGG-AAACCC
   consensus/100%                    ................................................................................
   consensus/90%                     ................................................................................
   consensus/80%                     uus.ss..ussss..ssssssssssssss..........sssss..s.ssssssusss..s.ssssss....s......s
   consensus/70%                     uuA.ss..uusAu..TsCCCsTsCCsGss..........sssCsssC.sCCsAuuuuu.sG.Gsusss....u.Ass.ss

                    cov    pid 961         .         .         .         0         .         .         .         . 1040
 1 BhEt_3         100.0% 100.0%      GTGGCGCCCACTCCTTCCGTGCCGTCACTGTTCCCGAGCTCACCCAGCAGATGTTCGACCCCAAGAACATGATGGCTGCT
 2 BeEt_2          60.7%  19.0%      --------------------------------------------------------------------------------
 3 BeEt_1          60.7%  19.7%      --------------------------------------------------------------------------------
 4 KhEt_1          82.5%  23.5%      TTGATGCAG---T---CGCAACCCTCGCCTCG-----CGG----------CGGACGACATCGAGGA--------------
```

```
  5 BeEt_9          94.5%  24.6%      TGGGAGGCA-GTT---CGCAAGCCT-TCAGCT-----CTTCGGCGGGCGGAC---CGACGTCAAGAA-CCGTTTGGTCCG
  6 SaEt_7          95.0%  23.9%      TGGGAGGGCAGGT---CGCTAGCCCCCTCAGT-----CCTTCGCGGCGGGACGAACGATCAAAGGAAGCGGATTGGTCGA
  7 SaEt_6          92.6%  24.3%      TGGGAGGGCAGGT---CGCTAGCCCCCTCAGT-----CCTTCGCGGCGGGACGAACGATCAAAGGAAGCGGATTGGTCGA
  8 SaEt_9          93.9%  23.7%      TGGAAGGCAGT-------CGCAGCCCATCAGC-----CTTCGGCGGCCGGACGGACGATCA-AGGAA-ACGATTGGTCGA
  9 BeEt_8          93.2%  22.9%      AGGCAGGTCGG-------C--AGCCCCTCAGC-----CTC-G-CGGCCGGA-CGACGATCAAAGGAAACGGTTTGGTCGA
 10 BeEt_6          82.5%  22.4%      GGAGGCAGTCG-------C--AGCCCCTCAGC-----CTC-G-GCGGCGG------------------------------
 11 BeEt_5          92.9%  24.2%      GGAGGCAGTCG-------C--AGCCCCTCAGC-----CTC-G-GCGGCGGAACGACGATCCAAGAAACCGGTTGGCGGAC
 12 BhEt_7          82.5%  24.1%      TGGAGGCAG---T---CGCAGCCCTCGGCCTC-----A--CGG--------CGGACGACGTCGAGGAC----CTGGTCGA
 13 BhEt_6          84.6%  23.2%      TGGAGGCAG---T---CGCAGCCCTCGGCCTC-----A--CGG--------CGGACGACGTCGAGGAC----CTGGTCGA
 14 BhEt_5          88.4%  24.2%      TGGAGGCAG---T---CGCAGCCCTCGGCCTC-----A--CGG--------CGGACGACGTCGAGGAC----CTGGTCGA
 15 BhEt_4          90.9%  24.9%      TGGAGGCAG---T---CGCAGCCCTCGGCCTC-----A--CGG--------CGGACGACGTCGAGGAC----CTGGTCGA
 16 KhEt_2          80.3%  24.2%      GATAGGTTT---T---CGTGATTG--------------------------------------------------------
 17 KaEt_1          61.0%  24.9%      --------------------------------------------------------------------------------
 18 KhEt_5          81.4%  25.4%      TGGAGGCAG---T---CGCACCCCTCGGCCTC-----A--CGG--------CG---------------------------
 19 KhEt_3          81.1%  24.6%      TGTAGGAGG---T---TCCATCCCTTGGCCT-------------------------------------------------
 20 KhEt_4          82.5%  25.1%      -GGAGGCAG---T---CGCAGCCCTCGGCCTC-----A--CGG--------CGGACGA-----------------------
 21 KaEt_3          68.2%  24.9%      --------------------------------------------------------------------------------
 22 BhEt_9          57.5%  24.6%      --------------------------------------------------------------------------------
 23 BhEt_8          59.9%  25.3%      --------------------------------------------------------------------------------
 24 KaEt_6          82.7%  23.9%      TGGTGCTTC---G---CAACCCTCGGCC---T-----C-GCGG--------CGGACGACATC------------------
 25 SaEt_5          91.0%  23.4%      TGGAGGCAG---T---CGCAACCCTCGGCCCT-----C-GCGG--------CGGACGACATCGAGGAC----GTTGTCGA
 26 KaEt_7          82.2%  24.1%      TGGAGGCAG---T---CGCAACCCTCGGCCTC-----G-CGGC--------GGAC-------------------------
 27 KaEt_4          82.4%  24.1%      TGGAGGCAG---T---CGCAACCCTCGGCCTC-----G-CGGC--------GGACG------------------------
 28 KaEt_9          77.2%  24.2%      TGG-----------------------------------------------------------------------------
 29 KaEt_8          81.9%  24.1%      TGGAGGCAG---T---CGCAACCCTCGGCCTC-----G-CGGC--------GG---------------------------
 30 BeEt_7          81.8%  23.9%      TGGAGGCAG---T---CGCAACCCTCGGCCTC-----G-CGGC--------G----------------------------
 31 BeEt_3          82.1%  23.7%      TGGAGCAGT-------CGCAACCCTCGGCCTC-----G-CGGC--------GGAC-------------------------
 32 KhEt_8          82.2%  23.8%      TGGAGCAGT-------CGCAACCCTCGGCCTC-----G-CGGC--------GGACG------------------------
 33 KhEt_7          78.5%  23.9%      TGGAGCAGT-------CG--------------------------------------------------------------
 34 BhEt_2          61.8%  22.7%      --------------------------------------------------------------------------------
 35 BhEt_1          52.4%  18.6%      --------------------------------------------------------------------------------
 36 BeEt_4          83.5%  26.0%      TGGATTCGT---T---CGGGTCCCTCGGCCTG-----GCGGCG--------G--AGGACATCAA-----------------
 37 SaEt_3          81.9%  25.0%      TGGATGCTG---T---CGCATCCCTCGGCCTC-----G------------------------------------------
 38 KhEt_9          82.7%  25.2%      TGGAGGCAG---T---CGCAGCCTTCGGGCCT-----CACGAC--------GGA--------------------------
 39 SaEt_4          82.7%  25.9%      TGGAGGCAG---T---CGCAGCCCTCGGCCTC-----GCGGCG--------CACGACAT---------------------
 40 SaEt_8          91.0%  25.3%      TGGAGGCAG---T---CGCAGCCCTCGGCCTC-----GCGACG----------CACAACGTCAAGGACC----TGGTCGA
 41 KaEt_5          91.7%  25.0%      TGGAGGCAG---T---CGCAGCCCCTCGGCCC-----TCGCGA--------CGCACAACGTCAATGGAC--CCTGGTCGA
 42 KaEt_2          82.2%  25.9%      TGGAGGCAG---T---CGCAGCCCTCGGCCTC-----GCGACG--------GACG-------------------------
 43 KhEt_6          82.5%  25.9%      TGGAGGCAG---T---CGCAGCCCTCGGCCTC-----GCGACG--------GACGA------------------------
 44 SaEt_2          70.8%  26.0%      --------------------------------------------------------------------------------
 45 SaEt_1          82.4%  25.9%      TGGAGGCAG---T---CGCAGCCCTCGGCCTC-----GCGACG--------GACG-------------------------
    consensus/100%                    ................................................................................
    consensus/90%                     ................................................................................
    consensus/80%                     .ss.............................................................................
    consensus/70%                     sGGusssss.........s..sssssssssss.....s..........................................

                   cov    pid 1041         :         .         .         .         .         1         .  ] 1114
  1 BhEt_3         100.0% 100.0%      TCCGACT-TCCGCAACG-----GTCGTTACCTGACCTGCTCTGCG-----------------------------
  2 BeEt_2          60.7%  19.0%      --------------------------------------------------------------------------
  3 BeEt_1          60.7%  19.7%      --------------------------------------------------------------------------
  4 KhEt_1          82.5%  23.5%      --------------------------------------------------------------------------
  5 BeEt_9          94.5%  24.6%      AACAAGCTTCAGGCACCCTCCAGATGTTAGTTGACCCCTTGGTCTCCAGGTCTGGGTTTTAACACGAGAGAGCT
  6 SaEt_7          95.0%  23.9%      CCAG-CATCAAGCCACCCATCAGCTGGTAGGTGCAACTGCCTTGGCGCTCCGA-GGCTGTGTTTACGTCAG---
  7 SaEt_6          92.6%  24.3%      CCAG-CATCAAGCCACCCATCAGCTGGTAG--------------------------------------------
  8 SaEt_9          93.9%  23.7%      CCAAGCATCCAGCCA-CCATCAAGTTGTAGTTGACCGCTTGGCCTAGTTGATGAGCCTGGATGTCT----G---
  9 BeEt_8          93.2%  22.9%      CAAGGCATCAGCAACTCATTCCAGCTGTAGTTGACTCCTTGGTCAGCTGTGTTGATA-----------------
 10 BeEt_6          82.5%  22.4%      --------------------------------------------------------------------------
 11 BeEt_5          92.9%  24.2%      CAAGGCTTCAAGCAACCCGTCAAGCGGTAAGTGACCCGTTGG--------------------------------
 12 BhEt_7          82.5%  24.1%      CCAGCTC----GGCACCCT---CAGTGTAGTTGACTCGTTGGCCAGTTT-GT--TTACCGGGA-----------
 13 BhEt_6          84.6%  23.2%      CCAGCTC----GGCACCCT---CAGTGTAGTTGACTCGTTGGCCAGTTT-GT--TTACCGGGA-----------
 14 BhEt_5          88.4%  24.2%      CCAGCTC----GGCACCCT---CAGTGTAGTTGACTCGTTGGCCAGTTT-GT--TTACCGGGA-----------
 15 BhEt_4          90.9%  24.9%      CCAGCTC----GGCACCCT---CAGTGTAGTTGACTCGTTGGCCAGTTT-GT--TTACCGGGA-----------
 16 KhEt_2          80.3%  24.2%      --------------------------------------------------------------------------
 17 KaEt_1          61.0%  24.9%      --------------------------------------------------------------------------
 18 KhEt_5          81.4%  25.4%      --------------------------------------------------------------------------
 19 KhEt_3          81.1%  24.6%      --------------------------------------------------------------------------
 20 KhEt_4          82.5%  25.1%      --------------------------------------------------------------------------
 21 KaEt_3          68.2%  24.9%      --------------------------------------------------------------------------
 22 BhEt_9          57.5%  24.6%      --------------------------------------------------------------------------
 23 BhEt_8          59.9%  25.3%      --------------------------------------------------------------------------
 24 KaEt_6          82.7%  23.9%      --------------------------------------------------------------------------
 25 SaEt_5          91.0%  23.4%      CAAGCTC----AGCTCCTT---CAGTGTAATGACCCT-TGGGCCAGTT-GT--TACCGAA--------------
 26 KaEt_7          82.2%  24.1%      --------------------------------------------------------------------------
 27 KaEt_4          82.4%  24.1%      --------------------------------------------------------------------------
 28 KaEt_9          77.2%  24.2%      --------------------------------------------------------------------------
 29 KaEt_8          81.9%  24.1%      --------------------------------------------------------------------------
 30 BeEt_7          81.8%  23.9%      --------------------------------------------------------------------------
 31 BeEt_3          82.1%  23.7%      --------------------------------------------------------------------------
```

```
32 KhEt_8          82.2%  23.8%      --------------------------------------------------------------------------
33 KhEt_7          78.5%  23.9%      --------------------------------------------------------------------------
34 BhEt_2          61.8%  22.7%      --------------------------------------------------------------------------
35 BhEt_1          52.4%  18.6%      --------------------------------------------------------------------------
36 BeEt_4          83.5%  26.0%      --------------------------------------------------------------------------
37 SaEt_3          81.9%  25.0%      --------------------------------------------------------------------------
38 KhEt_9          82.7%  25.2%      --------------------------------------------------------------------------
39 SaEt_4          82.7%  25.9%      --------------------------------------------------------------------------
40 SaEt_8          91.0%  25.3%      CGAGCTC----GGCACCCT---CAGTGTAGTG-ACCCCTTGGATCAGTT-TG--TGGTTATCACCGAGAAGGA-
41 KaEt_5          91.7%  25.0%      CGAGCAT----CGGCACCCTTCAGTTGTAGTTGACCCCTTGGGCCCAGA-TT--TGTTTTACCAGGAA------
42 KaEt_2          82.2%  25.9%      --------------------------------------------------------------------------
43 KhEt_6          82.5%  25.9%      --------------------------------------------------------------------------
44 SaEt_2          70.8%  26.0%      --------------------------------------------------------------------------
45 SaEt_1          82.4%  25.9%      --------------------------------------------------------------------------
   consensus/100%                    ..........................................................................
   consensus/90%                     ..........................................................................
   consensus/80%                     ..........................................................................
   consensus/70%                     ..........................................................................
```

MView 1.63, Copyright © 1997-2018 Nigel P. Brown
